# Superstructures of Organic–Polyoxometalate Co‐crystals as Precursors for Hydrogen Evolution Electrocatalysts

**DOI:** 10.1002/anie.202112298

**Published:** 2021-12-02

**Authors:** Shuang Li, Zhenyang Zhao, Tian Ma, Pradip Pachfule, Arne Thomas

**Affiliations:** ^1^ Functional Materials Department of Chemistry Technische Universität Berlin Hardenbergstraße 40 10623 Berlin Germany; ^2^ College of Polymer Science and Engineering Sichuan University Chengdu 610065 China

**Keywords:** 4d metals, carbides/nitrides, metal–organic precursors, organic-POM co-crystals, superstructures

## Abstract

Molybdenum‐based carbides and nitrides have been considered as catalysts for the hydrogen evolution reaction (HER). One of the challenges in using Mo‐based HER electrocatalysts is establishing well‐defined precursors which can be transformed into Mo‐based carbides/nitrides with controllable structure and porosity. We report the synthesis of a series of superstructures consisting of organic–polyoxometalate co‐crystals (O‐POCs) as a new type of metal–organic precursor to synthesize Mo‐based carbides/nitrides in a controlled fashion and to use them for efficient catalytic hydrogen production. This protocol enables to create electrocatalysts composed of abundant nanocrystallites and heterojunctions with tunable micro‐ and nanostructure and mesoporosity. The best performing electrocatalyst shows high HER activity and stability with a low overpotential of 162 mV at 100 mA cm^−2^ (in comparison to Pt/C with 263 mV), which makes it one of the best non‐noble metal HER catalysts in alkaline media and seawater.

## Graphical abstract

The synthesis of 2D mesoporous Mo carbides/nitrides for catalytic hydrogen production was realized by a series of organic–polyoxometalate co‐crystals (O‐POCs). These metal–organic precursors enable to create Mo‐based electrocatalysts composed of abundant nanocrystallites and heterojunctions with tunable micro‐ and nanostructure and mesoporosity, therefore resulting in high HER activity and stability in both alkaline condition and seawater.

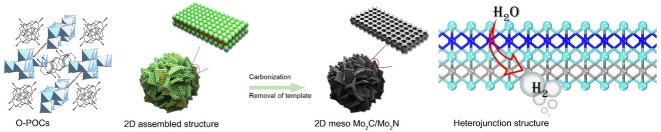

## Introduction

Hydrogen gas, a clean and sustainable energy source, has become one of the most promising alternatives for replacing fossil fuels.^[1]^ Electrocatalytic reduction of water to molecular hydrogen via the hydrogen evolution reaction (HER) provides a promising solution to produce cost‐effective hydrogen in high purity.^[2]^ However, the production of hydrogen utilizing water splitting requires highly efficient and robust catalytic materials.^[3]^ Although noble metals and their derivatives are the most active HER catalysts, their low stability, high cost, and scarcity significantly impede their commercial utilization in scalable hydrogen production.^[4]^ The investigation of cost‐effective and earth‐abundant materials that show high activity and stability has therefore attracted significant interest and became an essential target towards a future hydrogen economy.

Various classes of earth‐abundant transition‐metal compounds have been developed for HER catalysis.^[5]^ In course of these studies, experimental and theoretical investigations have suggested that *4d*‐metal compounds, such as Mo carbides/nitrides are promising for HER catalysis due to their similar electronic properties to the Pt‐group metals.^[6]^ However, the catalytic activity of current reported *4d*‐metal carbide/nitride‐based catalysts are still far behind the Pt‐based catalysts, which might result from the so far applied synthetic methods, yielding low structural control, thus Mo‐based compounds with large particle size, small surface area and low Mo content. For example, Mo carbides/nitrides prepared by reaction of Mo oxides with a mixture of hydrogen gas and carbon/nitrogen‐containing gases (CH_4_, C_2_H_6_, CO, or NH_3_) usually exhibit low surface areas and porosities, due to the inevitable particle coalescence/aggregation, which severely inhibits their catalytic performances.^[7]^ Therefore, a controllable synthesis of micro‐ and nanostructured *4d*‐metal carbides/nitrides with high porosity and abundant active *4d*‐metal compounds is urgently needed to develop efficient HER catalysts.

Recently, the synthesis of nanostructured carbides/nitrides was realized by carbonizing metal‐organic frameworks (MOFs) as inorganic‐organic hybrid precursors.^[8]^ However, due to the difficulties in synthesizing *4d*‐metal‐nodes, Mo‐metal carbides/nitrides have only been obtained when loading polyoxometalates into MOFs, for example, a H_3_PMo_12_O_40_ loaded Cu‐MOF^[9]^ or H_3_PMo_12_O_40_ loaded MIL‐100 (Fe).^[10]^ However, the fabricated Mo‐based carbides/nitrides are usually randomly distributed on a carbon matrix with relatively low Mo content (<40 wt. %).^[11]^ The performance of current carbon supported *4d*‐metal carbides/nitrides is indeed largely hindered by the low loading amount of active metal species in the catalysts. Therefore, an efficient way to directly create a high Mo‐metal containing metal‐organic precursor with defined nanostructure and superstructure to obtain metal carbides and nitrides with extreme high molybdenum loading is still challenging but of great importance to develop novel, noble metal free catalysts for HER.

## Results and Discussion

**Figure 1:**
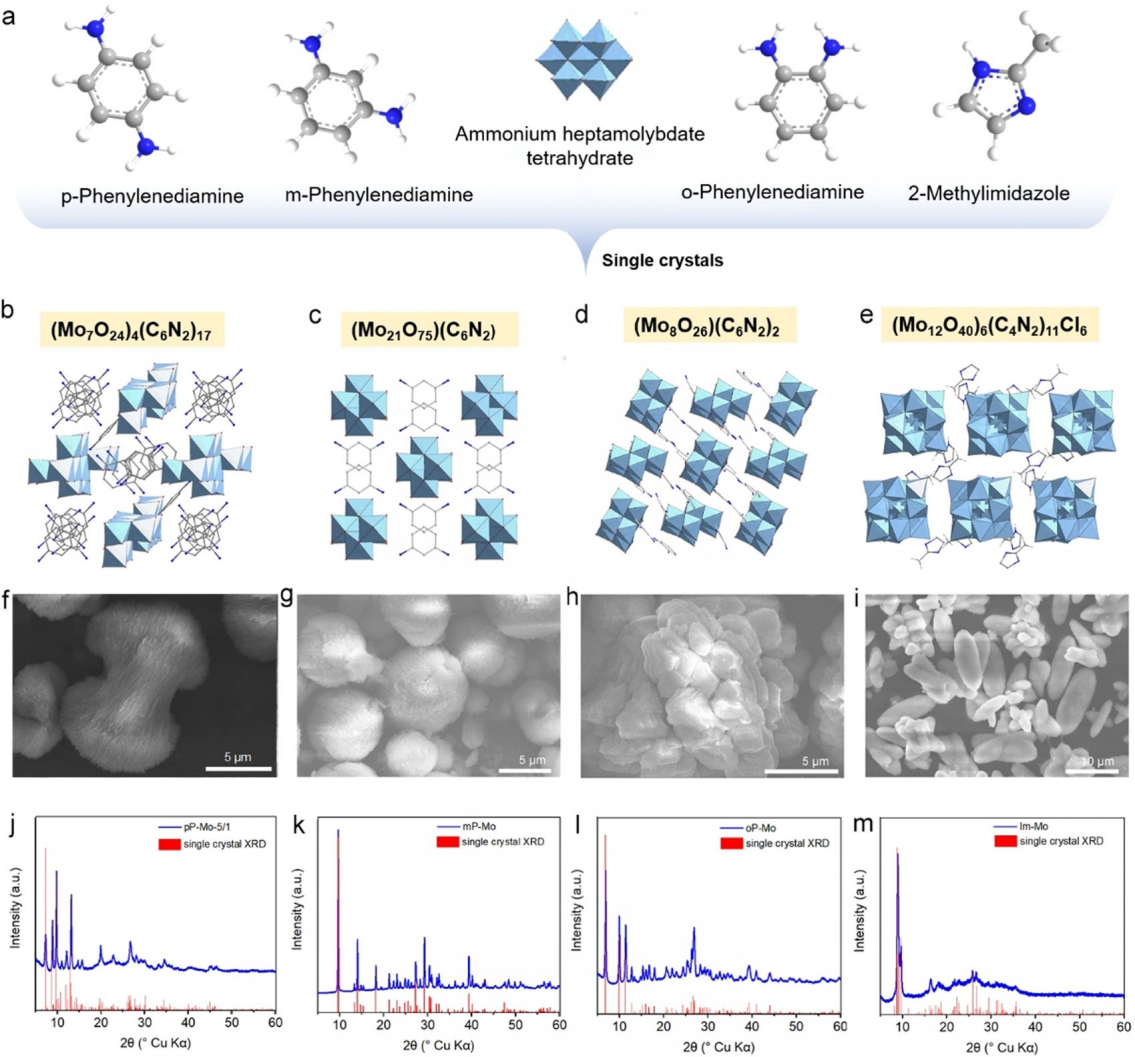
a) Molecular structures of *p*‐phenylenediamine, *m*‐phenylenediamine, Mo_7_O_24_ cluster, *o*‐phenylenediamine, and *2*‐methylimidazole. b)–e) Single‐crystal structures of the obtained O‐POCs from different organic molecules. f)–i) SEM images of the O‐POCs‐silica composites. j)–m) Simulated XRD patterns from single‐crystal structures and the powder XRD of the precursors: j) pP‐Mo‐5/1, k) mp‐Mo, i) oP‐Mo, and m) Im‐Mo.

Herein, a series of organic‐polyoxometalate co‐crystals (O‐POCs) as a new type of highly tunable metal‐organic precursor is presented to synthesize Mo‐based carbides/nitrides for efficient hydrogen production. Ammonium molybdate was applied as precursor for the Mo‐based polyoxometalate, nitrogen‐containing molecules (*p*‐phenylenediamine, *m*‐phenylenediamine, *o*‐phenylenediamine, and *2*‐methylimidazole) as organic ligands and silica colloids as nuclei for crystallization and hard templates to form stable O‐POCs‐silica composites (Figure 1 a). The inorganic and organic compounds were dissolved in an aqueous silica nanoparticle dispersion in different amounts and diluted hydrochloric acid (1 M) was added to the mixture to precipitate the O‐POCs. The as‐synthesized precursors were named pP‐Mo, mP‐Mo, oP‐Mo, and Im‐Mo depending on the used organic linker. Additional experiments on pP‐Mo precursors with different ratio of acid reveal the acid induced assembly process during the precipitation (details in the Supporting Information). After careful structural analysis, these composites were carbonized in an Argon oven at different temperatures and finally the silica was removed to yield mesoporous Mo_2_C/Mo_2_N electrocatalysts (for details on the synthesis, see the SI).

The O‐POCs created from the four different ligands with ammonium heptamolybdate were characterized by single‐crystal X‐ray diffraction (Figure 1 b–e, Figures S1–S4), revealing a stoichiometry of (Mo_7_O_24_)_4_(*p*‐phenylenediamine)_17_ for pP‐Mo, (Mo_21_O_75_)(*m*‐phenylenediamine) for mP‐Mo, (Mo_8_O_26_)(*o*‐phenylenediamine)_2_ for oP‐Mo, and (Mo_12_O_40_Cl)_6_(*2*‐methylimidazole)_11_ for Im‐Mo. In the presence of silica, the O‐POCs assemble into superstructures, for example, pP‐Mo/silica shows a flower‐like architecture composed of nanosheets, mP‐Mo/silica microspheres from aggregated nanoblocks, while oP‐Mo/silica exhibit a rather undefined microstructure assembled again from nanosheets. In contrast, the Im‐Mo/silica shows a spindle‐like microstructure also composed of aggregated, yet less‐defined nanoparticles (Figure 1 f–i, Figures S5–S8). The powder X‐ray diffraction (XRD) patterns of the obtained precursors are shown in Figure 1 j–m, where all the patterns are fitting well with the simulated patterns from single crystal XRD indicating that the powder O‐POCs have the same molecular ratios and crystal structures as shown in Figure b‐e. pP‐Mo was chosen as representative precursor for further investigation since the pP‐Mo‐derived catalysts exhibit the best HER catalytic performance (see above). The catalysts obtained from the other O‐POCs are presented in the supporting information. If not otherwise specified, materials described in the following are derived from pP‐Mo as O‐POCs precursor.

**Figure 2:**
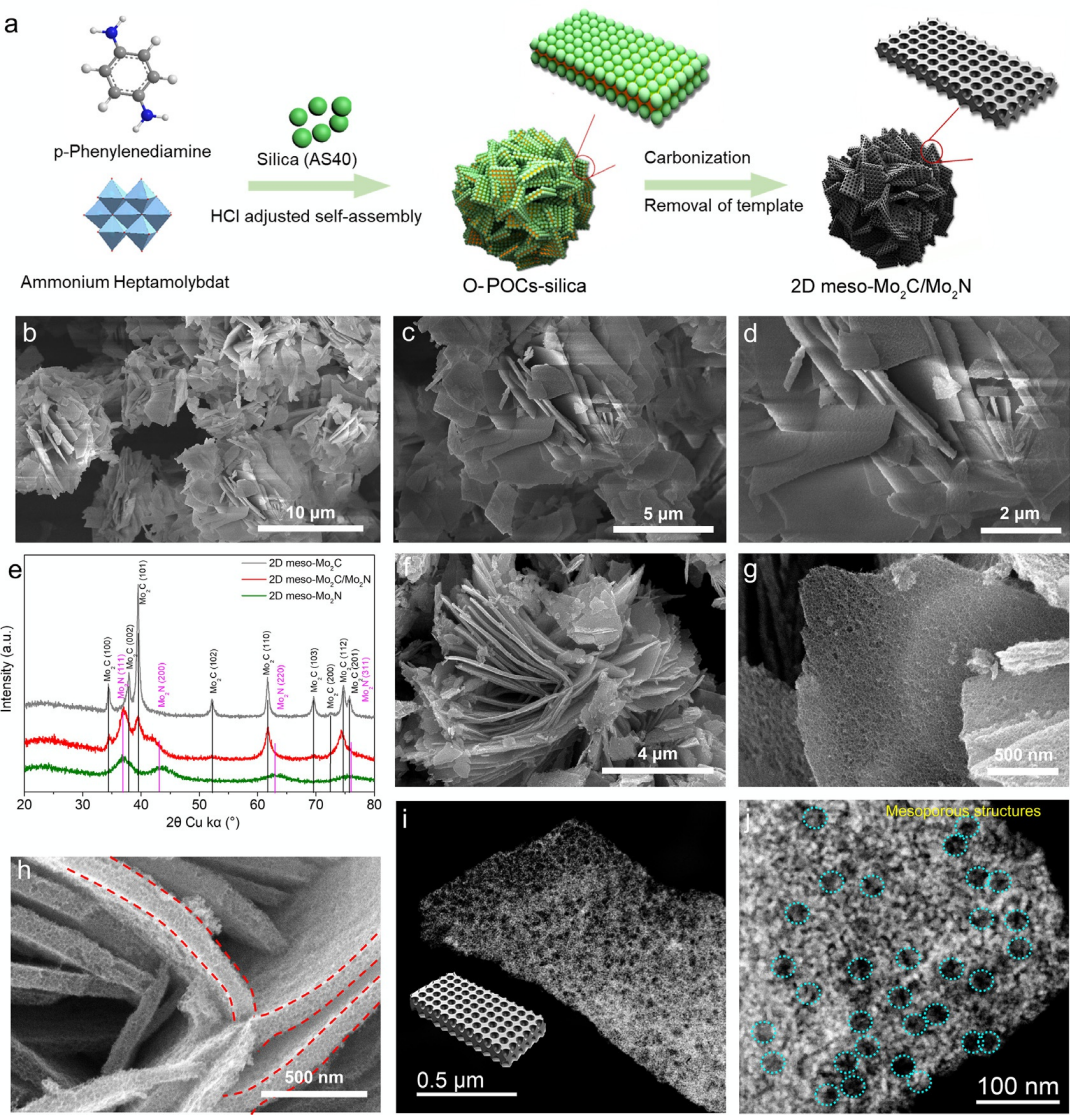
a) Illustration of the synthesis procedure; b)–d) SEM images of the pP‐Mo‐1/3 precursor, e) powder XRD of carbonized pP‐Mo‐1/3 treated at different temperatures; f)–h) SEM images of the obtained 2D meso‐Mo_2_C/Mo_2_N; i, j) HAADF‐STEM images of 2D meso‐Mo_2_C/Mo_2_N (circles show mesopores).

Further pP‐Mo‐based O‐POCs‐silica composites were synthesized with different amount of acid to reveal the changes of assembled morphologies. Scanning electron microscopy (SEM) show that the structure of the primary crystallites assembling the microstructures change gradually from nanofibers to nanosheets with increasing amount of acid and uniform 2D nanosheets can be obtained for a molar ratio of pP and HCl of 1:3 (Figure 2 a–d, Figures S9, S10), which was the ratio used for further studies. X‐ray photoelectron spectroscopy (XPS) analysis of the pP‐Mo obtained with different acid ratio shows similar elemental compositions of C, N, O, Mo, and Si (Figure S11). The Mo3d and N1s region analysis shows that the increasing amount of acid changes the amount of protonated nitrogen species in the structure as well as the content of Mo‐oxygen clusters (Figures S12, S13). This can also be confirmed by comparing the single‐crystal structure of pP‐Mo‐5/1, with a composition (Mo_7_O_24_)_4_(*p*‐phenylenediamine)_17_ to the structure of pP‐Mo‐1/3 with a composition of (Mo_8_O_28_)_3_(*p*‐phenylenediamine)_10_, showing the depletion of organic moieties within the co‐crystals at decreasing pH, proposedly because the organic counter cations are progressively replaced by hydronium ions, which effects the composition and structure of the co‐crystals. The 2D pP‐Mo composite was then carbonized in an Ar atmosphere at different temperatures (700, 800, and 900 °C) and the silica subsequently removed by NH_4_HF_2_ treatment. The powder XRD of pP‐Mo carbonized at different temperatures and after removal of silica (pP‐Mo‐700, pP‐Mo‐800, and pP‐Mo‐900) show characteristic peaks that can be indexed to Mo_2_N and Mo_2_C, respectively, as shown in Figure 2 e. Notably, the pP‐Mo‐700 exhibits just 4 broad peaks, which can be attributed to Mo_2_N (111), Mo_2_N (200), Mo_2_N (220), and Mo_2_N (311) (Figure 2 e), indicating the formation of only Mo_2_N at the temperature of 700 °C, consequently the sample was named 2D meso‐Mo_2_N. Meanwhile, all diffraction peaks for pP‐Mo‐900 fit well to Mo_2_C, indicating the formation of Mo_2_C at 900 °C (2D meso‐Mo_2_C). Finally, mixed phases of Mo_2_N and Mo_2_C can be obtained at a temperature of 800 °C (2D meso‐Mo_2_C/Mo_2_N).

The morphology of the 2D meso‐Mo_2_C/Mo_2_N was investigated by SEM, which shows that the nanosheet morphology is well maintained after carbonization (Figure 2 f). Additionally, abundant uniform and interconnected mesopores can be observed within the sheets at higher resolution (Figure 2 g). The cross‐section view shows that the porous nanosheets all have a thickness of ≈40 nm (Figure 2 h). The control sample, which was synthesized without silica nanoparticles, show nanosheets as well but without any visible mesoporous structure (Figure S14). 2D meso‐Mo_2_C/Mo_2_N was further investigated by high‐angle annular dark‐field scanning transmission electron microscopy (HAADF‐STEM) (Figure 2 i, j). As seen in the HAADF‐STEM images, the abundant bright nanodots indicate the high amount of Mo‐based species distributed on the surface of 2D meso‐Mo_2_C/Mo_2_N. The mesoporous structure of the material is confirmed by N_2_ adsorption/desorption measurements showing a type IV isotherm (with a Brunauer–Emmett–Teller (BET) surface area of 74.2 m^2^ g^−1^). The pore size distribution was calculated from the adsorption branch of the isotherms by the QSDFT model for cylinder/sphere pores, which show uniform mesopores with a size of ≈29 nm, which matches well with the particle size of silica nanoparticles used as template (Figure S15).

**Figure 3:**
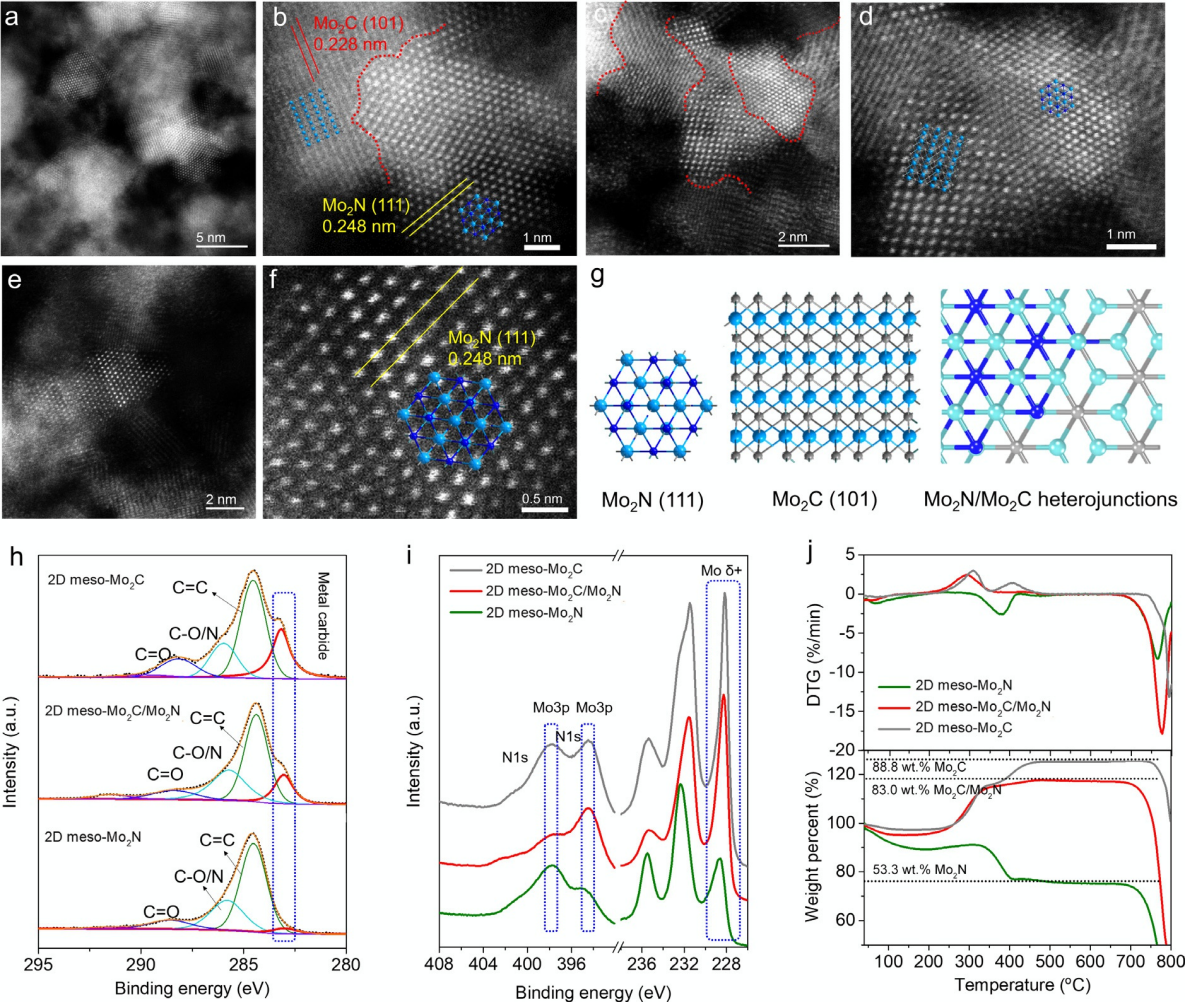
a)–f) High‐resolution HAADF‐STEM images of the Mo_2_C, Mo_2_N nanocrystals, and their heterojunctions in the 2D meso‐Mo_2_C/Mo_2_N; g) theoretical crystal structures of Mo_2_N, Mo_2_C and Mo_2_C/Mo_2_N heterojunctions; h),i) high‐resolution XPS peaks of h) C1s, i) Mo3d, Mo3p, and N1s; j) TGA and corresponding DTG curves.

To further investigate the structure of the 2D meso‐Mo_2_C/Mo_2_N composite, high‐resolution HAADF‐STEM has been performed. As shown in Figure 3, there are two types of Mo‐based crystalline nanoparticles distributed around the pore structures with abundant crystal boundaries. Particles with a lattice distance of about 0.228 nm, matching the (101) facet of Mo_2_C and with a lattice distance of about 0.248 nm that matches the (111) facet of Mo_2_N nanocrystals are observed (Figure 3 b, S16), which is consistent with the XRD results. Notably, a large number of small interconnected Mo_2_C/Mo_2_N crystals can be observed, suggesting the formation of Mo_2_C/Mo_2_N heterojunctions (Figure 3 c). Figure 3 d–f shows that the atomic arrays in the crystal structures can be well‐matched with the simulated crystal structures of Mo_2_C (101) and Mo_2_N (111) (Figure 3 g). Heterojunction catalysts have been reported to show better activity than single‐component catalysts, which was attributed to the different electronic properties of the Mo atoms at the heterojunction interface and the maximized synergistic effects between the two components.^[12]^

XPS was conducted to further analyze the composition and chemical state of the 2D meso‐Mo_2_N, 2D meso‐Mo_2_C/Mo_2_N, and 2D meso‐Mo_2_C. First, the survey spectra show clear peaks corresponding to C, N, O, and Mo in the materials (Figure S17). The elemental content derived from these measurements (Table S1) shows an increasing amount of Mo with increasing carbonization temperature. The highest content of Mo with 62.2 wt. % is observed for 2D meso‐Mo_2_C. Furthermore, the N amount is decreasing from 8.8 wt. % for 2D meso‐Mo_2_N to 3.3 wt. % (2D meso‐Mo_2_C/Mo_2_N) and 1.5 wt. % (2D meso‐Mo_2_C), indicating the decreasing amount of Mo_2_N species. High‐resolution XPS scans of C1s, Mo3d, and N1s were conducted for all samples to investigate structural changes after treatment of the precursor at different temperatures. The C1s peak which can be assigned to C in a metal carbide at 283.0 eV increased with the temperature, showing the ongoing formation of Mo_2_C (Figure 3 h). Other carbon peaks can be attributed to adventitious carbon. Figure 3 i shows the N1s, Mo3p, and Mo3d peaks of the materials. The formation of Mo_2_C and Mo_2_N can be confirmed via the emerging peaks of Mo^δ+^ at binding energies of 228.4 eV. Compared with 2D meso‐Mo_2_N, the 2D meso‐Mo_2_C/Mo_2_N and 2D meso‐Mo_2_C exhibit a much stronger Mo^δ+^ peak intensity, as shown in Figure 3 i, which has been reported as the most active Mo site compare with higher valent Mo.^[13]^ The N1s region scan can be separated into two parts: one is overlapping with the Mo3p peak at 394.5 and 398.2 eV, which again shows an increase of intensity with rising temperature. The other is the N1s peak at ≈397.2 eV indicating the formation of metal nitrides and as expected the 2D meso‐Mo_2_N shows the highest peak intensity at this region.

Thermogravimetric analysis (TGA) and the derivative thermogravimetry (DTG) measurements in O_2_ atmosphere of all materials show the oxidation of Mo_2_N or Mo_2_C to Mo oxides between 300–450 °C, causing an increase of weight percent (Figure 3 j). The sharp decrease of weight at around 700 °C, can be attributed to the sublimation of MoO_3_. The content of Mo_2_N or Mo_2_C in the materials was calculated based on both TGA and XPS; the result shows a rising Mo content with increasing temperature. Thus, 2D meso‐Mo_2_C/Mo_2_N (83.0 wt. %) and 2D meso‐Mo_2_C (88.8 wt. %) show much higher Mo contents than 2D meso‐Mo_2_N (53.3 wt. %), which can be attributed to the higher carbon residues at lower carbonization temperature. The results calculated from TGA are slightly higher than observed from XPS measurements, since XPS is a surface sensitive method, still the observed trends of Mo content is the same (Table S1).

**Figure 4:**
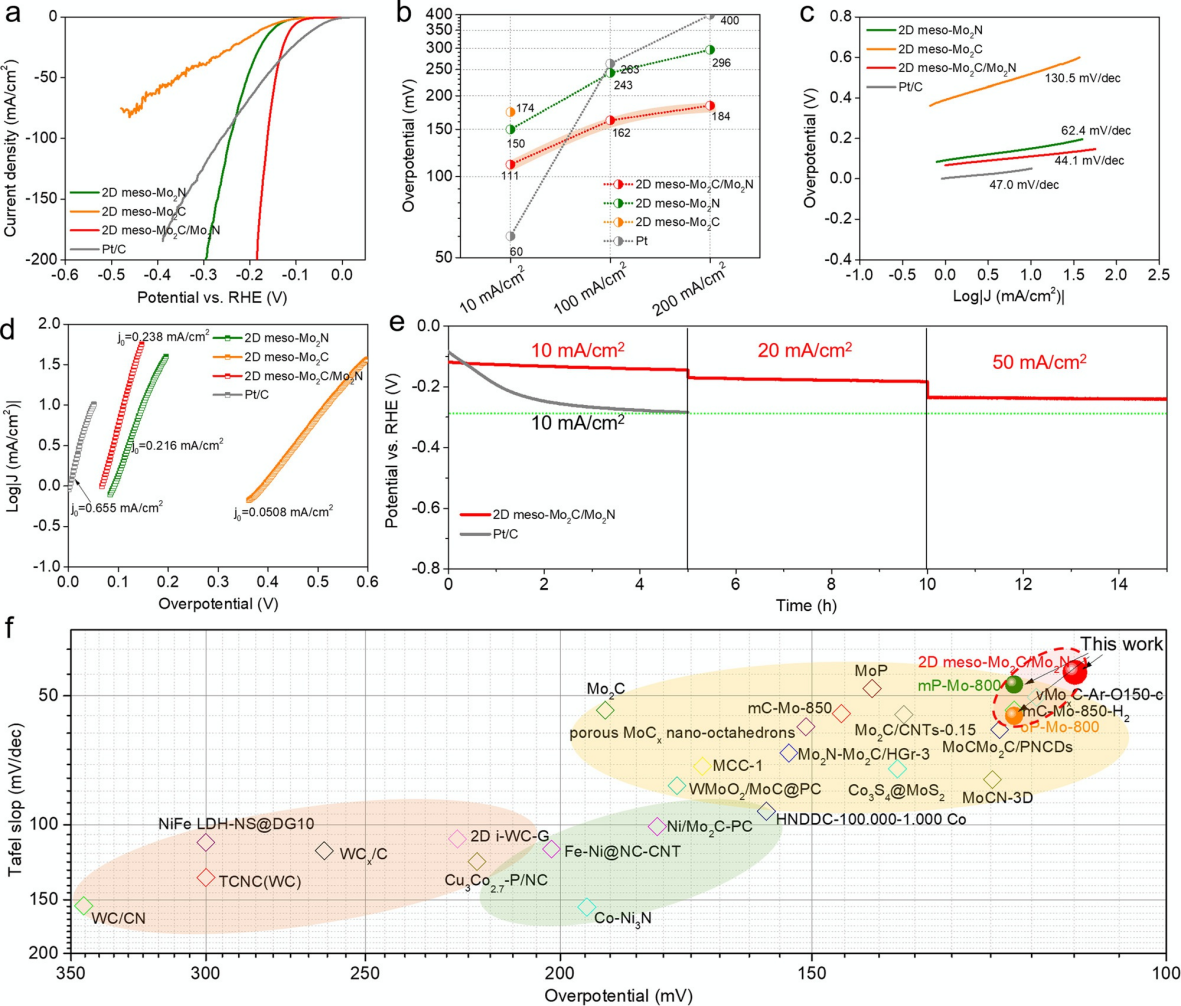
a) Polarization curves, b) overpotential at different current densities, c) Tafel plots, and d) the exchange current density of 2D meso‐Mo_2_N, 2D meso‐Mo_2_C/Mo_2_N, 2D meso‐Mo_2_C, and Pt/C; e) Longtime stability of the 2D meso‐Mo_2_C/Mo_2_N and Pt/C catalysts working at current densities of 10, 20, and 50 mA cm^−2^, and f) the comparison of different Mo,W, and other transition metal based catalysts in terms of Tafel slope and overpotential at the current density of 10 mA cm^−2^.

To assess the HER electrocatalytic activity in alkaline media, the prepared mesoporous catalysts, control samples and commercial Pt/C are evaluated in 1 M KOH solutions with a mass loading of 0.38 mg cm^−2^ on a glassy carbon disk electrode. The activities of all catalysts were evaluated by polarization curves and corresponding Tafel plots (Figure 4, Figure S18–20). As shown in Figure 4 a, the 2D meso‐Mo_2_N and 2D meso‐Mo_2_C catalysts without a heterojunction structure show similar overpotentials of 150 mV and 174 mV to reach the current density of 10 mA cm^−2^, while a much lower overpotential at a current density of 100 mA cm^−2^ was observed for 2D meso‐Mo_2_N (243 mV) compared to 2D meso‐Mo_2_C (higher than 500 mV). This can be attributed to the higher carbon contents in the matrix providing better conductivity. The 2D meso‐Mo_2_C/Mo_2_N shows a much improved HER activity with lower overpotentials of 111 mV and 162 mV at the current densities of 10 and 100 mA cm^−2^, respectively. The 2D meso‐Mo_2_C/Mo_2_N catalyst outperforms Pt/C at an overpotential of 125 mV with a rapidly rising cathodic current. At the overpotential of 184 mV, 200 mA cm^−2^ current density can be reached by the 2D meso‐Mo_2_C/Mo_2_N catalyst. Notably, the 2D meso‐Mo_2_C/Mo_2_N catalyst also showed a much‐enhanced performance compared with the nonporous catalysts synthesized without silica nanoparticles (Figure S18). The significantly improved performance of current densities for HER can be attributed to the intrinsic activity improvement from the heterojunction structures, where interfacial Mo atoms are bonded to both, C and N, resulting in an optimal surface electronic structure for H_2_O dissociation and proton adsorption and the significant mass transfer improvement provided by the mesopores within the 2D meso‐Mo_2_C/Mo_2_N catalyst. Furthermore, the Mo_2_C/Mo_2_N synthesized from different precursors at 800 °C (pP, mP, and oP) all show good activity for HER (Figure S20) except the materials prepared with Im ligands, probably due to the faster decomposition of Im ligands and its higher nitrogen content, which yield preferable formation of Mo_2_N. This can be confirmed by the XRD measurement of Im‐Mo‐800 (Figure S21), which shows only the main peaks of Mo_2_N rather than a Mo_2_C/Mo_2_N heterojunction structure. The Tafel plots indicate that the 2D meso‐Mo_2_C/Mo_2_N catalyst presents a much smaller Tafel slope of 44.1 mV/decade than 2D meso‐Mo_2_N (62.4 mV/decade) and 2D meso‐Mo_2_C (130.5 mV/decade) catalysts (Figure 4 b), verifying its efficient kinetics in H_2_ evolution. In addition, the Tafel slope of the 2D meso‐Mo_2_C/Mo_2_N catalyst is also smaller than that of commercial Pt/C (47.0 mV/decade). The current exchange density (*j*_0_), which reflects the intrinsic HER catalytic activity, has been calculated from the polarization curves (Figure 4 a). The *j*_0_ value for 2D meso‐Mo_2_C/Mo_2_N catalyst (0.238 mA cm^−2^) and 2D meso‐Mo_2_N catalyst (0.216 mA cm^−2^) is much higher than for other reported Mo/W carbide‐based catalysts, (Figure 4 d, Figure S22). To better understand the activities of each active site, the mass activity and turn over frequency (TOF) values of these catalysts were calculated based on the Mo content from XPS measurements (Figure S23). Notably, the 2D meso‐Mo_2_C/Mo_2_N catalyst shows a mass current of 60 A/g_Mo_ and TOF value of 0.030 H_2_ s^−1^ per Mo atom, which is more than 8 or 15 times higher than the values for 2D meso‐Mo_2_N and 2D meso‐Mo_2_C, respectively, indicating much higher activity of Mo in heterojunction structures.

As 2D meso‐Mo_2_C/Mo_2_N shows the highest HER activity, its stability for continuous production of H_2_ was measured and compared with Pt/C (Figure 4 e). With constant current densities of 10, 20, and 50 mA cm^−2^, the overpotentials only slightly increased after running for 5 h for each step, i.e., only a 25 mV shift was observed for 10 mA cm^−2^, 13 mV for 20 mA cm^−2^, and 5 mV for 50 mA cm^−2^ (Figure 4 e). In contrast, the overpotential of Pt/C catalysts shifted 201 mV after running for 5 h at 10 mA cm^−2^. Recently reported activities of Mo,W, and other transition metal based catalysts are summarized and compared in Figure 4 f in terms of Tafel slope vs. overpotential at 10 mA cm^−2^, demonstrating that the 2D meso‐Mo2C/Mo2N catalyst shows an exceptional activity compared to other Mo‐based catalysts.[^13^, ^14^]

**Figure 5:**
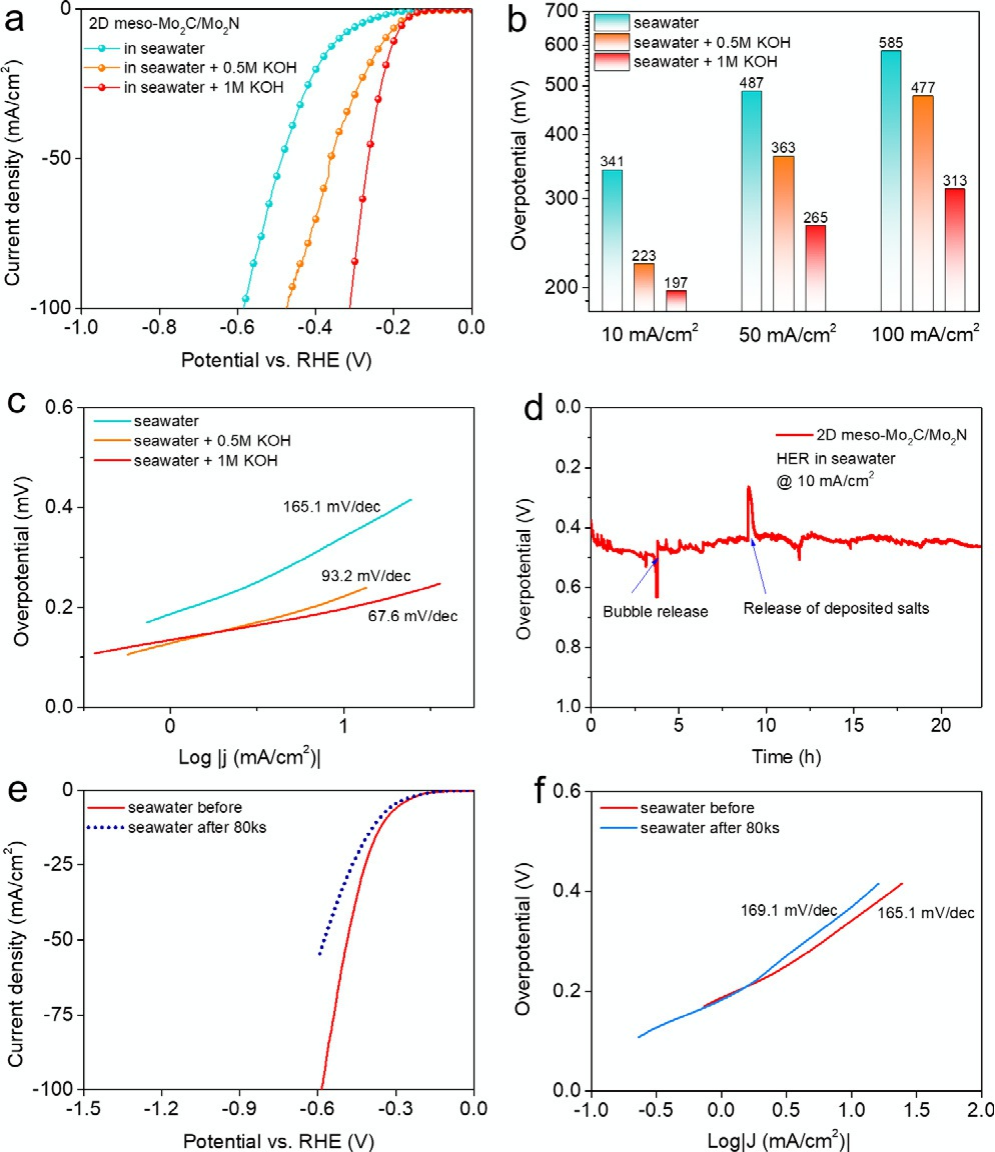
a) Polarization curves, b) overpotential at different current densities, and c) Tafel plots of 2D meso‐Mo_2_C/Mo_2_N catalyst in seawater with different amounts of KOH; d) Longtime stability of the 2D meso‐Mo_2_C/Mo_2_N catalyst working in artificial seawater at current densities of 10 mA cm^−2^, and e) the corresponding polarization curves before and after stability test and f) the corresponding Tafel plot.

Based on the above observations, 2D meso‐Mo_2_C/Mo_2_N catalyst is a highly efficient electrocatalyst in alkaline electrolytes. Additionally, the Mo‐based carbides and nitrides are reported to have high corrosion resistance due to the high valence state of their Mo atoms, making them promising candidates for seawater electrolysis. As expected, the 2D meso‐Mo_2_C/Mo_2_N catalyst exhibit good HER performance in artificial seawater, requiring an overpotential of 341 mV to produce a current density of 10 mA cm^−2^ (Figure 5 a). This overpotential is lower than for most reported non‐noble metal catalysts for seawater HER (Table S2). By introducing different amounts of KOH in the artificial seawater, the HER overpotential of the 2D meso‐Mo_2_C/Mo_2_N catalyst can be further decreased from 341 to 197 mV (Figure 5 b), and the Tafel slope can be decreased from 165.1 to 67.6 mV dec^−1^ (Figure 5 c). More importantly, stable hydrogen production from artificial seawater can be achieved for more than 20 h at the current density of 10 mA cm^−2^ (Figure 5 d) with 2D meso‐Mo_2_C/Mo_2_N, which is a large improvement compared to Pt/C (which is retaining only 5.5 % of its catalytic current density after only 4 h). The polarization curves of 2D meso‐Mo_2_C/Mo_2_N catalyst before and after the stability test and the corresponding Tafel slop also indicates the good stability of the catalyst in seawater condition (Figure 5 e,f).

## Conclusion

The synthesis of 2D meso‐Mo_2_C/Mo_2_N nanosheets was successfully achieved using newly designed O‐POCs precursors. The nitrogen and carbon‐rich Mo precursors with well‐defined crystal structures ensure the formation of both Mo_2_N and Mo_2_C heterojunction structures. The silica nanoparticles not only act as nucleation site for the formation of O‐POCs structures and template during the carbonization process, but also prevent the aggregation of Mo species to form larger crystals during the carbonization process ensuring the formation of small Mo‐based nanocrystals. The 2D meso‐Mo_2_C/Mo_2_N catalyst exhibit excellent HER performance in various electrolytes and also in seawater. The material combines high activity and good stability, demonstrating the possibility of using 2D metal carbides and nitrides for electrocatalytic processes in harsh environments. Overall, the here shown approach not only creates a new pathway for the controllable synthesis of micro‐/nanostructured Mo‐based catalysts but also for the production of highly tunable metal‐organic materials and precursors for a broad range of applications.

## Conflict of interest

The authors declare no conflict of interest.

## Supporting information

As a service to our authors and readers, this journal provides supporting information supplied by the authors. Such materials are peer reviewed and may be re‐organized for online delivery, but are not copy‐edited or typeset. Technical support issues arising from supporting information (other than missing files) should be addressed to the authors.

Supporting Information
