# Supplementary material for: Superstructures of Organic–Polyoxometalate Co‐crystals as Precursors for Hydrogen Evolution Electrocatalysts
Source: Angew Chem Int Ed Engl. 2021 Dec 2;61(3):e202112298. doi: 10.1002/anie.202112298 (PMC9300107; doi:10.1002/anie.202112298)
Supplement: Supplementary file 1 — Supporting Information [file ANIE-61-0-s001.pdf]

Angewandte Chemie
Eine Zeitschrift der Gesellschaft Deutscher Chemiker

Supporting Information

Superstructures of Organic–Polyoxometalate Co-crystals as Precursors for Hydrogen Evolution Electrocatalysts

Shuang Li, Zhenyang Zhao, Tian Ma, Pradip Pachfule, and Arne Thomas**

anie_202112298_sm_miscellaneous_information.pdf

Table of Contents

1, Experimental details
2, Figure S1-S23
3, Table S1
4, Author contributions

Experimental Procedures

Chemical Reagents and Materials

Pt-carbon, Nafion solution, $H_{24}Mo_7N_6O_{24}\cdot 4H_2O$, p-Phenylenediamine, HCl (37%), NaCl, $MgCl_2$, $MgSO_4$, $CaCl_2$, $NaHCO_3$, Na_2SO_4, and KCl were obtained from Alfa Aesar. Monodisperse silica nanoparticles (22 nm, Ludox AS-40) and NH_4HF were purchased from Sigma Aldrich. Unless otherwise e stated, all the reagents were of analytical grade and were used as received. All aqueous solutions were prepared with DI water.

Characterizations and Methods

Scanning electron microscope (SEM): The morphology of the prepared precursors and carbons were observed by high-resolution FE-SEM (Hitachi S-4000 and Hitachi S-4800). All the carbonized materials were observed directly without gold coating. For the non-conductive precursors, the gold coating was deposited with a layer of about 1 nm.

Transmission electron microscopy (TEM): High angle annular dark-field scanning TEM (HAADF-STEM) was performed on a probe-corrected JEM ARM 200F S/TEM (JEOL Japan).

X-ray photoelectron spectra (XPS): XPS was measured on K-Alpha™ + X-ray Photoelectron Spectrometer System (Thermo Scientific) with Hemispheric 180 ° dual-focus analyzer with 128-channel detector. X-ray monochromator is Micro-focused Al-Kα radiation. For the measurement, the prepared powder samples were pressed and loaded on carbon taps, then pasted onto the sample holder for measurement. The data was collected with an X-ray spot size of 400 μm, 20 scans for the survey, and 50 scans for the regions.

Surface area and pore size distribution analysis: N_2 sorption analysis was conducted on a Quantachrome Quadrasorb SI instrument. All samples were degassed at 150 °C overnight before actual measurement. The surface area was calculated by using Brunauer-Emmett-Teller (BET) calculations. The pore size distribution (PSD) plot was recorded from the adsorption branch of the isotherm based on the QSDFT model for spherical pore.

Thermogravimetric analysis (TGA): TGA was conducted in O_2 conditions from room temperature to 800 ^oC with a ramp of 10 ^oC/min.

X-ray diffraction (XRD): Powder XRD of all the prepared samples were measured with the same condition on a Bruker D8 Advance instrument with Cu Kα radiation (λ=1.54 Å) at a generator voltage of 40 kV and a generator current of 50 mA. The single crystals were measured on single crystal XRD (Oxford Diffraction XCalibur).

Experimental details

Synthesis of the pP-Mo POCs structures. A schematic illustration of the synthesis of POM-organic single crystals is shown in Fig. 1A. Ammonium molybdate tetrahydrate ($(NH_4)_6Mo_7O_{24}\cdot 4H_2O$) is used as the Mo-POM precursor, while water is used as the solvent, and p-Phenylenediamine is selected as the organic agent. First, 10 g colloidal silica solution (Ludox AS-40) was diluted to 50 mL, 1mL 1M HCl was then added to adjust the pH value of the solution to ~2. Then, 1.08 g *p*-phenylenediamine was dissolved in 50 mL H_2O and mixed with the colloidal silica solution. 2.48 g $(NH_4)_6Mo_7O_{24}\cdot 4H_2O$ was also dissolved in 50 mL water and dropped into the above solution. Finally, different amounts of 1 M HCl (0, 4, 9, 14, and 19 mL) were added into the system to investigate possible changes in the morphologies of the assembled co-crystal structures. After reaction for 2 h, the products were collected by filtration and washed with H_2O and ethanol three times.

Synthesis of the mP-Mo POCs structures. A schematic illustration of the synthesis of POM-organic single crystals is shown in Fig. 1A. Ammonium molybdate tetrahydrate ($(NH_4)_6Mo_7O_{24}\cdot 4H_2O$) is used as the Mo-POM precursor, while water is used as the solvent, and m-Phenylenediamine is selected as the organic agent. First, 10 g colloidal silica solution (Ludox AS-40) was diluted to 50 mL, 1mL 1M HCl was then added to adjust the pH value of the solution. Then, 1.08 g *m*-phenylenediamine was dissolved in 50 mL H_2O and mixed with the colloidal silica solution. 2.48 g $(NH_4)_6Mo_7O_{24}\cdot 4H_2O$ was also dissolved in 50 mL water and dropped into the above solution. Finally, 14 mL 1 M HCl was added to the system. After reaction for 2 h, the product was collected by filtration and washed with H_2O and ethanol three times.

Synthesis of the oP-Mo POCs structures. A schematic illustration of the synthesis of POM-organic single crystals is shown in Fig. 1A. Ammonium molybdate tetrahydrate ($(NH_4)_6Mo_7O_{24}\cdot 4H_2O$) is used as the Mo-POM precursor, while water is used as the solvent,

and o-Phenylenediamine is selected as the organic agent. First, 10 g colloidal silica solution (Ludox AS-40) was diluted to 50 mL, 1 mL 1 M HCl was then added to adjust the pH value of the solution. Subsequently, 1.08 g o-Phenylenediamine was dissolved in 50 mL H_2O and mixed with the colloidal silica solution. 2.48 g $(NH_4)_6Mo_7O_{24}\cdot4H_2O$ was also dissolved in 50 mL water, then dropped into the above solution. Finally, 34 mL 1 M HCl was added to the system. After reaction for 2 h, the product was collected by filtration and washed with H_2O and ethanol three times.

Synthesis of the Im-Mo POCs structures. A schematic illustration of the synthesis of POM-organic single crystals is shown in Fig. 1A. Ammonium molybdate tetrahydrate ($(NH_4)_6Mo_7O_{24}\cdot4H_2O$) is used as the Mo-POM precursor, while water is used as the solvent, and 2-methylimidazole is selected as the organic agent. First, 10 g colloidal silica solution (Ludox AS-40) was diluted to 50 mL, 1mL 1 M HCl was then added to adjust the pH value of the solution. Then, 0.82 g 2-methylimidazole was dissolved in 50 mL H_2O and mixed with the colloidal silica solution. 2.48 g $(NH_4)_6Mo_7O_{24}\cdot4H_2O$ was also dissolved in 50 mL water and dropped into the above solution. Finally, 34 mL 1 M HCl was added to the system. After reaction for 2 h, the product was collected by filtration and washed with H_2O and ethanol three times.

Synthesis of the 2D mesoporous molybdenum carbides/nitrides: The obtained pP-Mo-1/3 were carbonized in Argon atmosphere under different temperatures (700, 800, and 900 ^oC), then the products were stirred with 4 M NH_4HF for 12 h, following by washing with H_2O and ethanol for 5 times by centrifugate.
The pP-Mo-5/1, pP-Mo-1/1, pP-Mo-1/2, and pP-Mo-1/4 were also carbonized under 800 ^oC, and then treated with the same washing procedure for comparison.
The mP-Mo, oP-Mo, and Im-Mo were also carbonized under 800 ^oC, and treated with the same washing procedure for comparison.

Electrochemical Measurements
Ink preparation. The catalyst ink was prepared by blending the catalyst powder (15 mg) with 100 µL Nafion solution (5 wt%) and 900 µL ethanol in an ultrasonic bath. 5 µL of catalyst ink was then pipetted onto the GC surface, leading to a catalyst loading of 0.38 mg/cm^2. Commercially available 20 wt% platinum on Vulcan carbon black (Pt/C from the Fuel Cell store) was measured with the same loading amount for comparison.

Electrodes and measurements. All the electrochemical measurements were carried out in a conventional three-electrode cell using the Gamry reference 600 workstation (Gamry, USA) at room temperature. RHE electrode and graphite rod were used as reference and counter electrodes, respectively. A glassy carbon (GC) RDE electrode with an area of 0.196 cm^2 served as the substrate for the working electrode to evaluate the HER activities of various catalysts. The electrochemical experiments were conducted in Ar saturated various electrolytes. The RDE measurements were conducted at a rotating speed of 1600 rpm with a sweep rate of 10 mV/s.

Artificial seawater preparation: 26.73 g of NaCl, 2.26 g of $MgCl_2$, 3.25 g of $MgSO_4$, 1.12 g of $CaCl_2$, 0.19 g of $NaHCO_3$, 3.48 g of Na_2SO_4, and 0.72 g of KCl were dissolved in ultrapure water with the volume of 1 L.
0.5 M KOH + Artificial seawater preparation: 100 mL 1 M KOH and 100 mL artificial seawater were mixed together to get the 0.5 M KOH + artificial seawater.
1 M KOH + Artificial seawater preparation: 200 mL artificial seawater was mixed with 0.2-mole KOH powder to form the 1 M KOH + artificial seawater.

Results and Discussion

Supplementary Figures

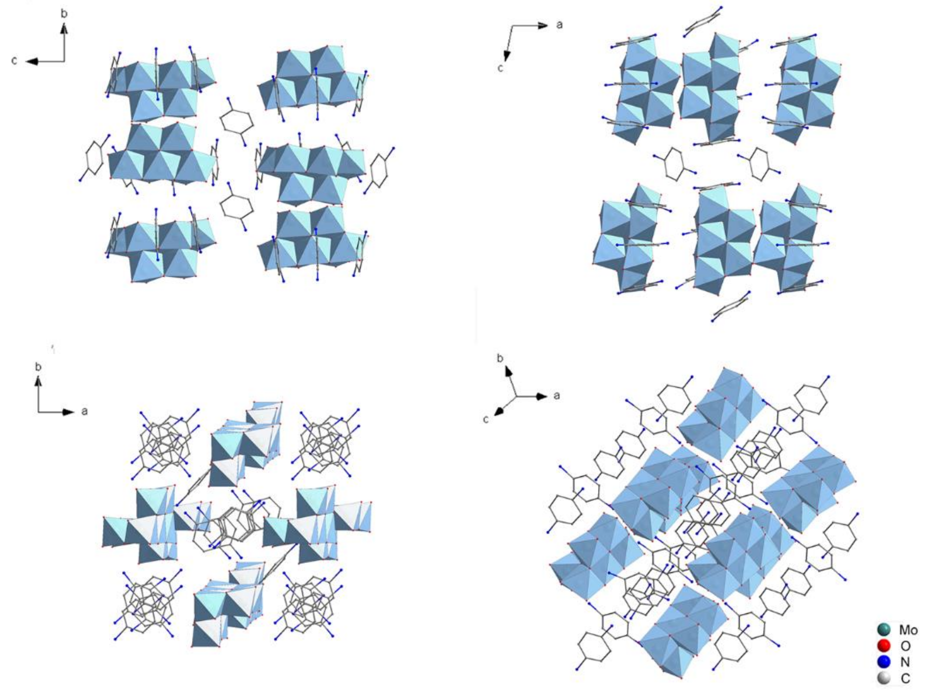

Figure S1. single crystal structure of pP-Mo-5/1 in directions of 100, 010, 001, and 111

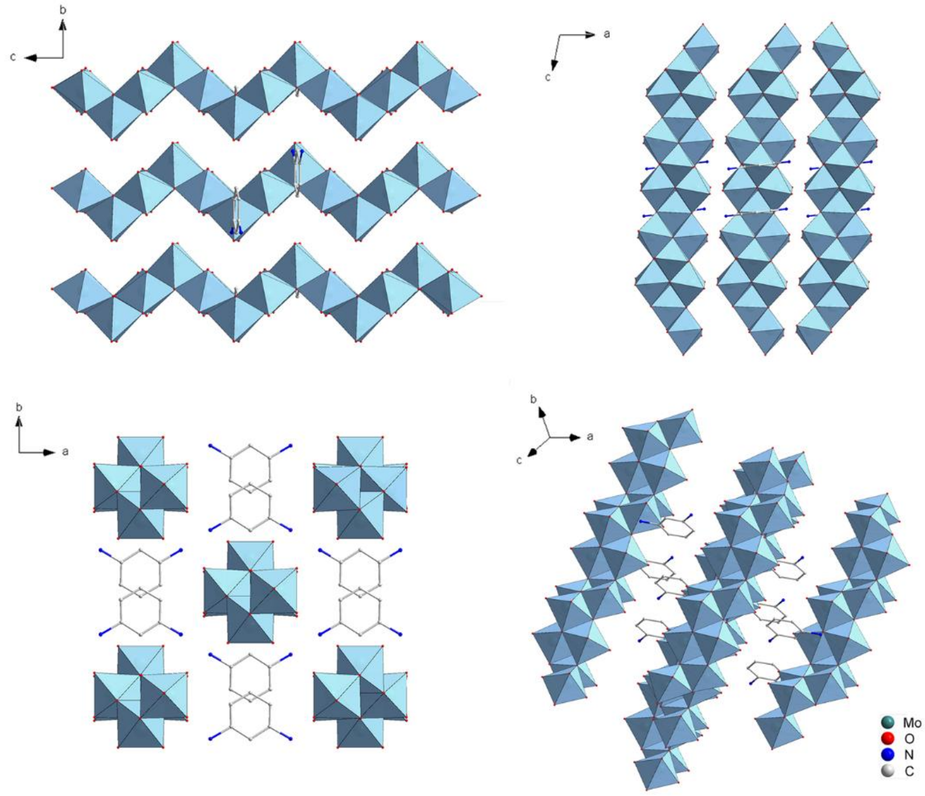

Figure S2. single crystal structure of mP-Mo in directions of 100, 010, 001, and 111

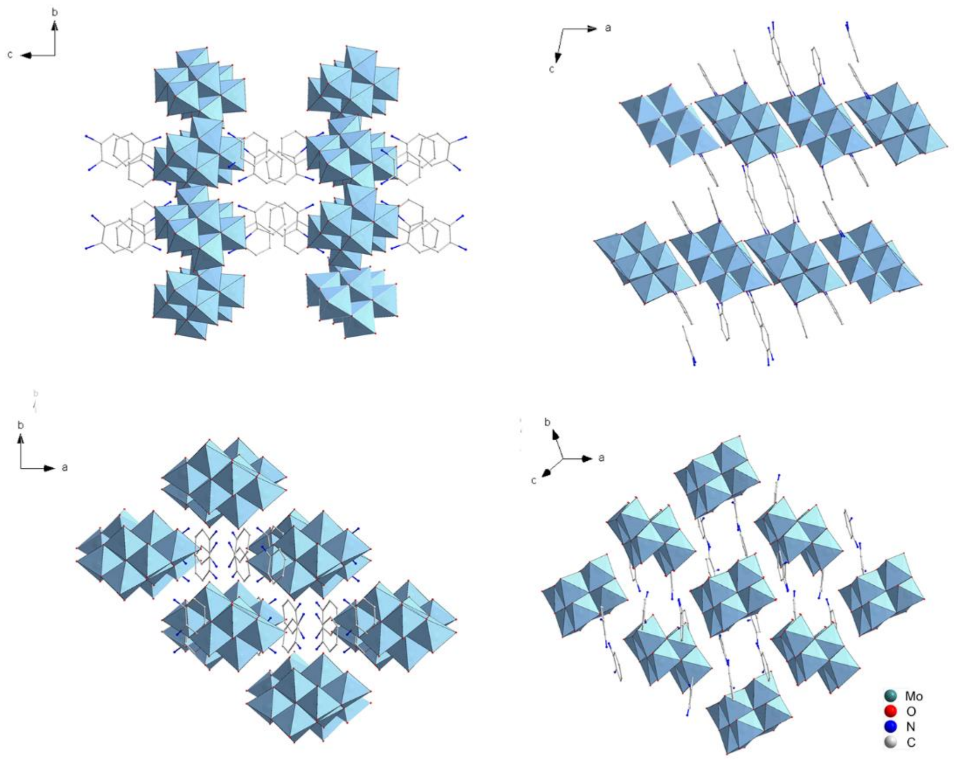

Figure S3. single crystal structure of oP-Mo in directions of 100, 010, 001, and 111

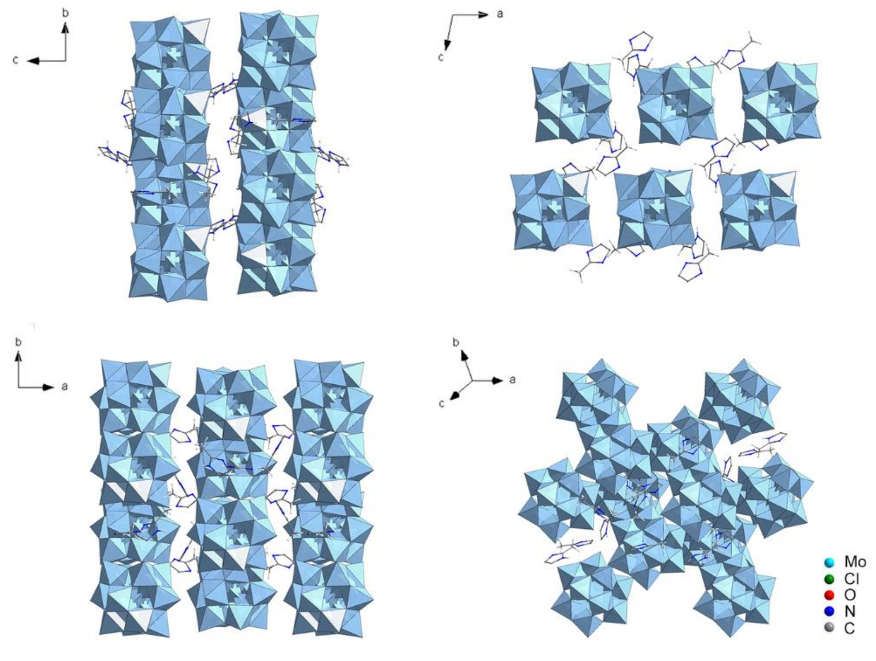

Figure S4. single crystal structure of Im-Mo in directions of 100, 010, 001, and 111

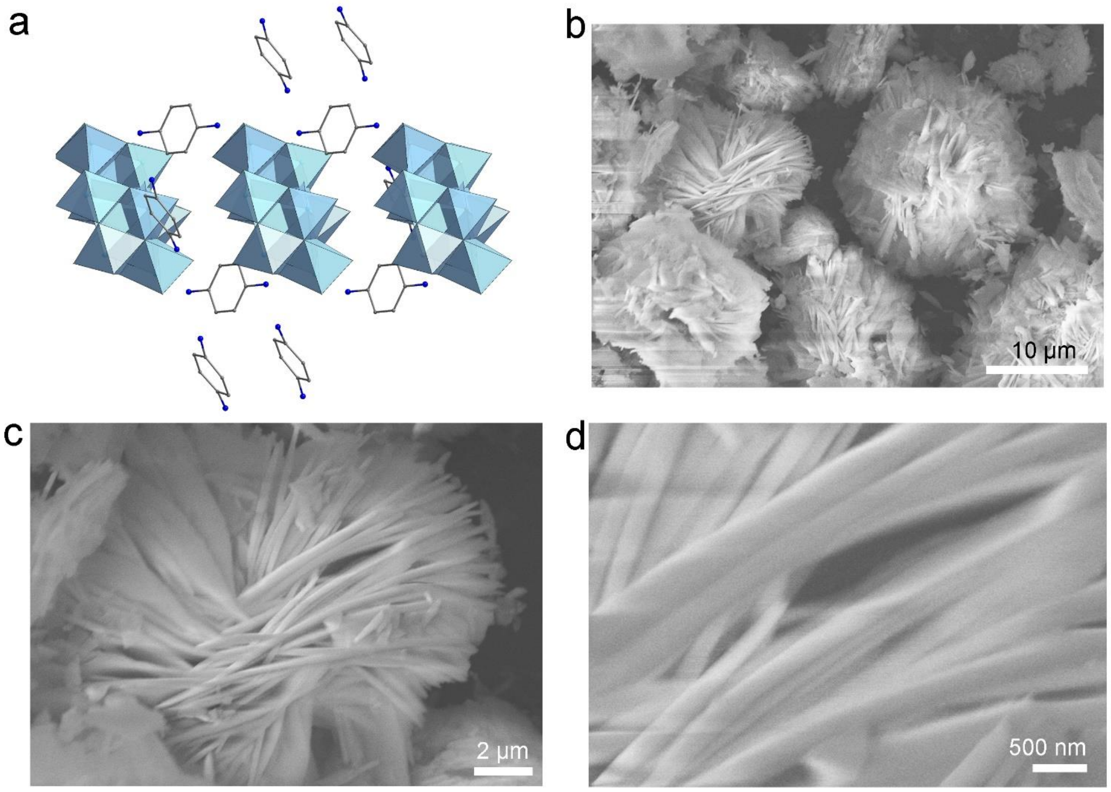

Figure S5. a) single crystal structure, b-d) SEM images of the pP-Mo-1/3.

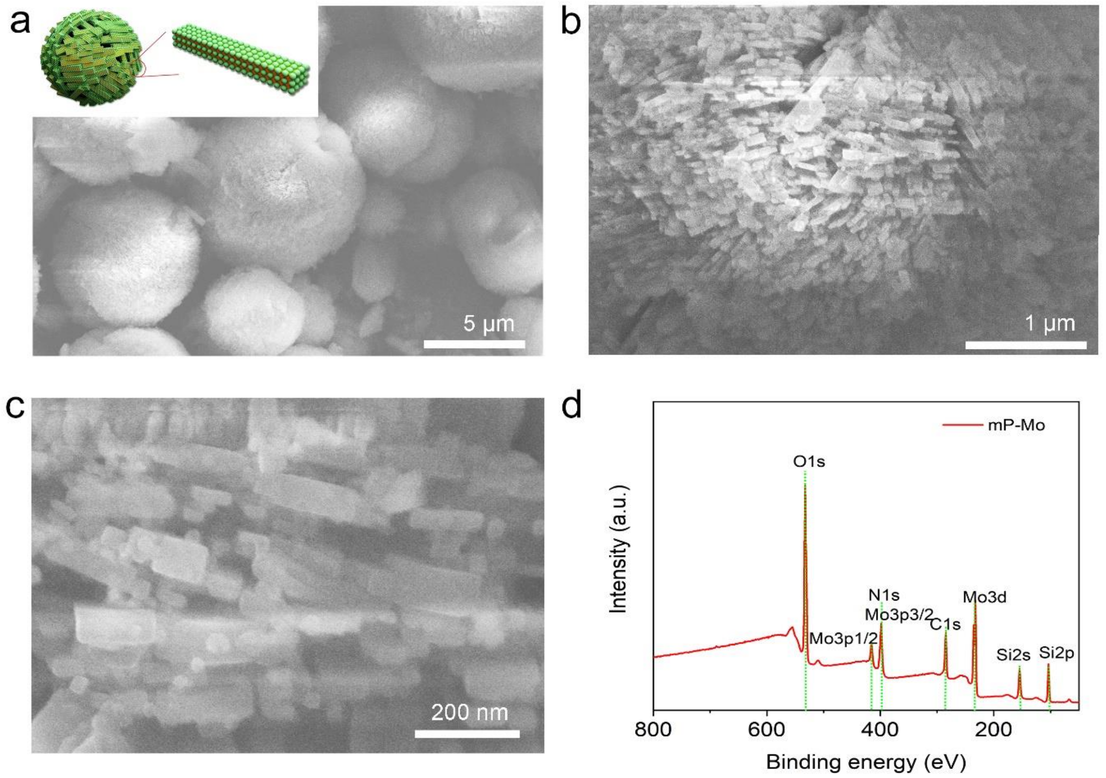

Figure S6. a-c) SEM images of mP-Mo structures, d) XPS survey spectra of the mP-Mo precursor.

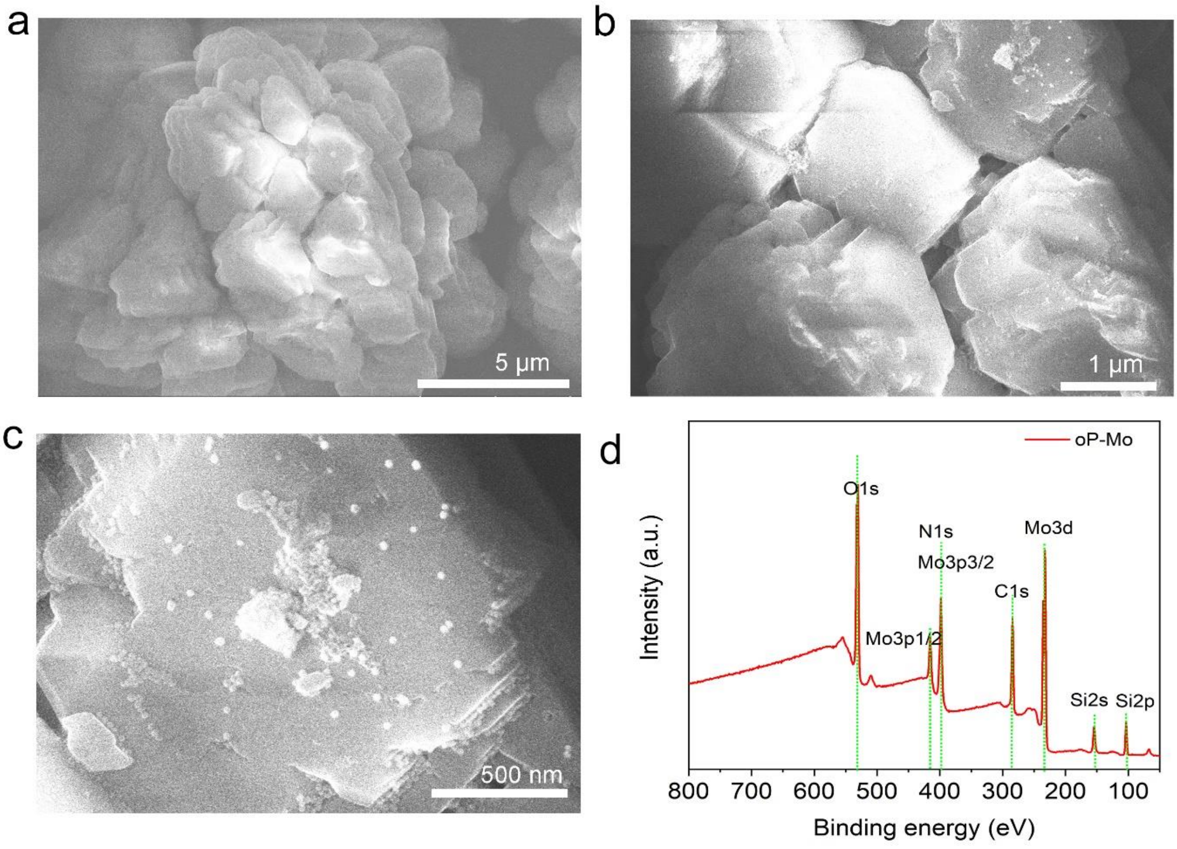

Figure S7. a-c) SEM images of oP-Mo structures, d) XPS survey spectra of the oP-Mo precursor.

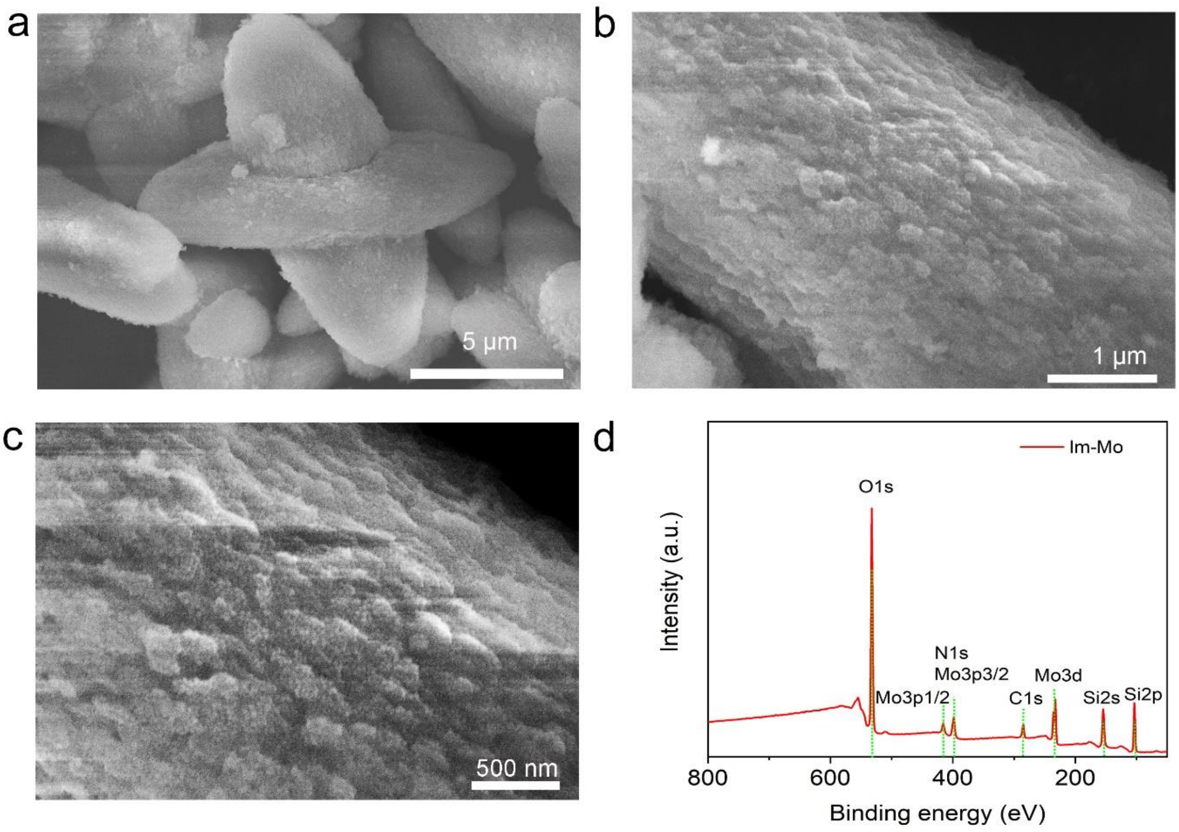

Figure S8. a-c) SEM images of Im-Mo structures, d) XPS survey spectra of the Im-Mo precursor.

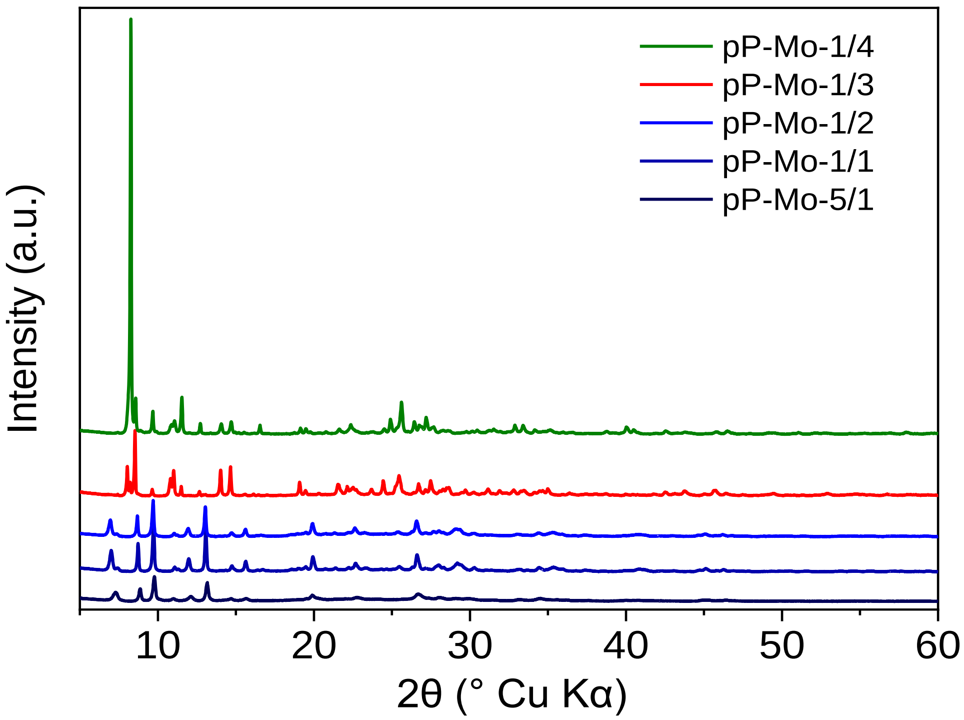

Figure S9. XRD patterns of products obtained from different acid amounts.

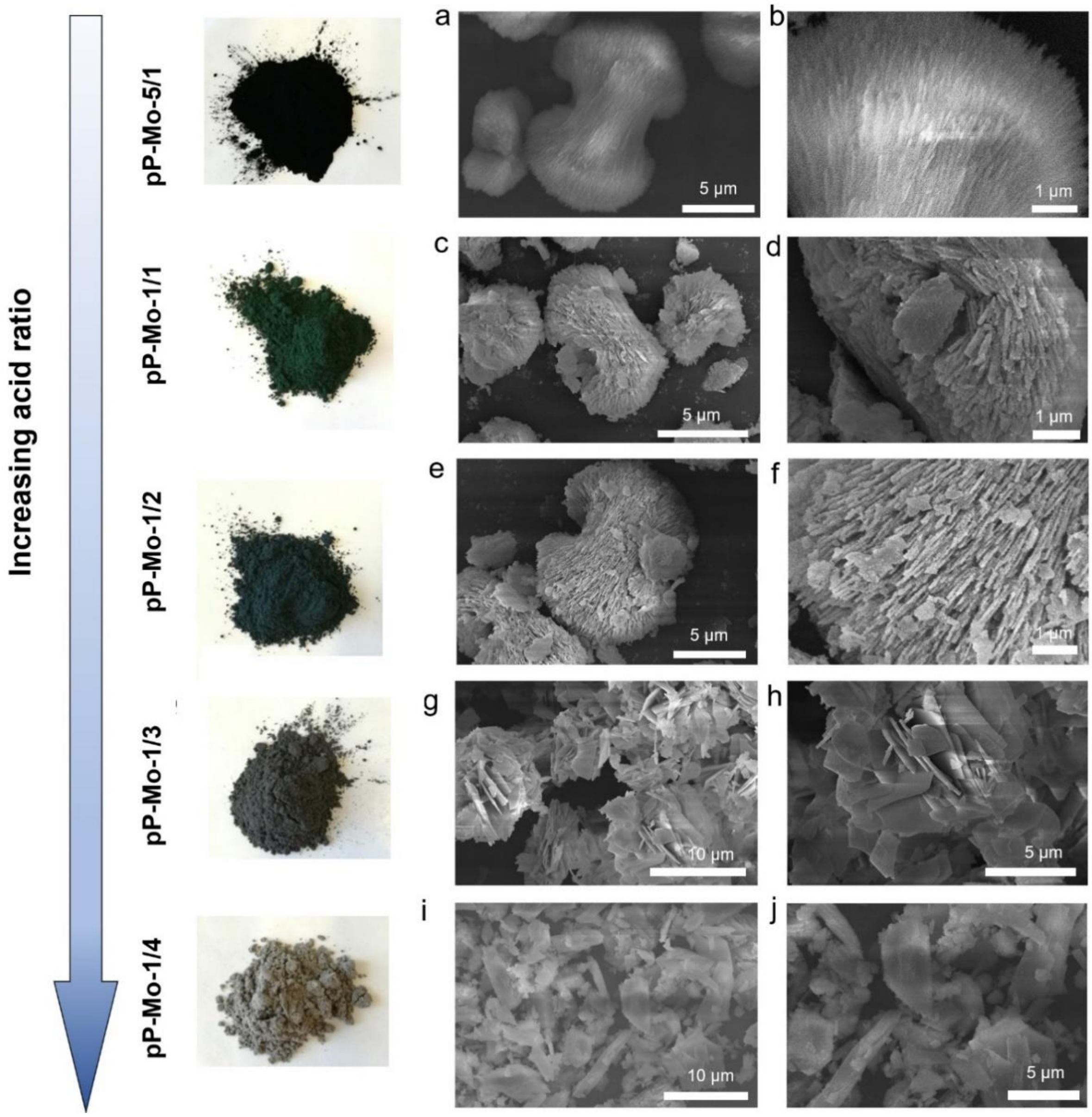

Figure S10. Photographs of products obtained from different acid amounts and SEM images of a, b) pP-Mo-5/1, c, d) pP-Mo-1/1, e, f) pP-Mo-1/2, g, h) pP-Mo-1/3, and i, j) pP-Mo-1/4. (The amount of acid was changed according to the molar ratio between pP and HCl from 5:1, 1:1, 1:2, 1:3, to 1:4)

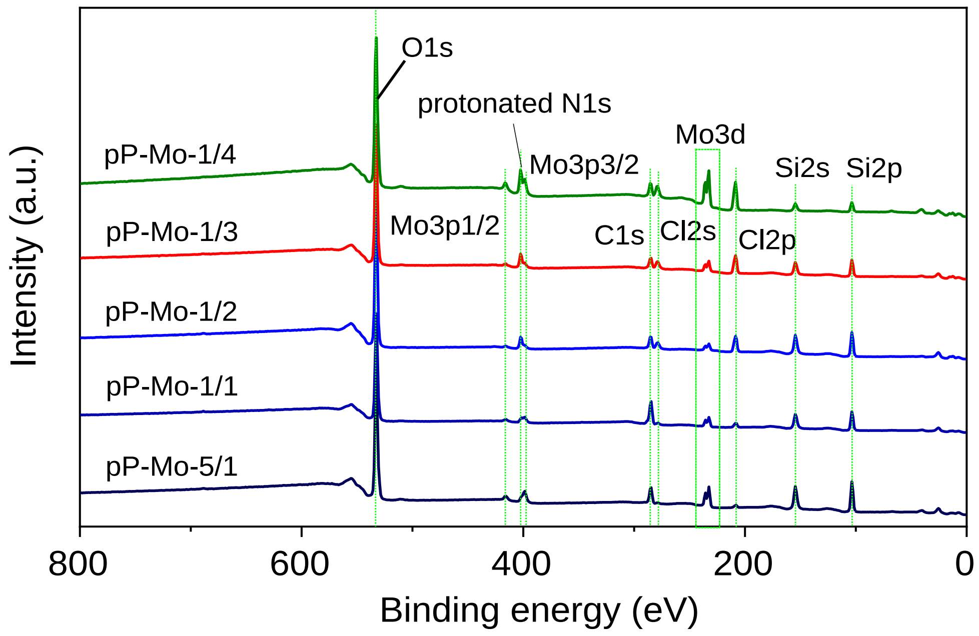

Figure S11. XPS survey spectra of pP-Mo precursors obtained from different acid ratios.

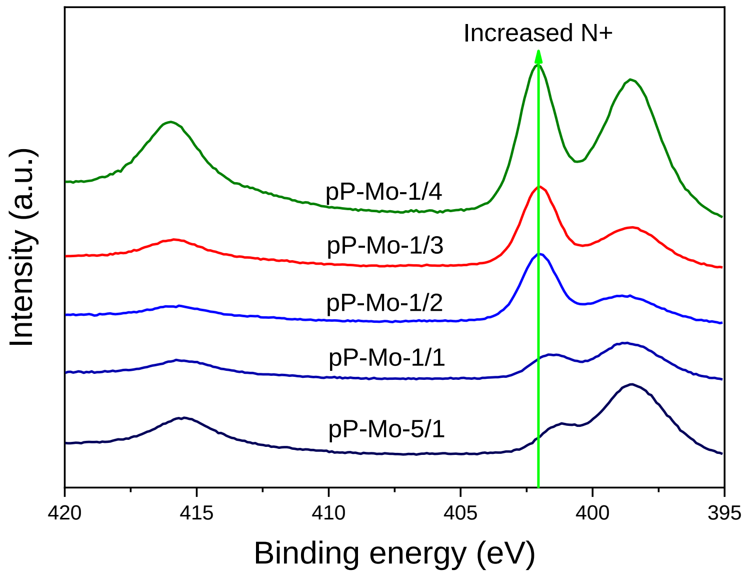

Figure S12. N1s and Mo3p region scan of pP-Mo precursors obtained from different acid ratios.

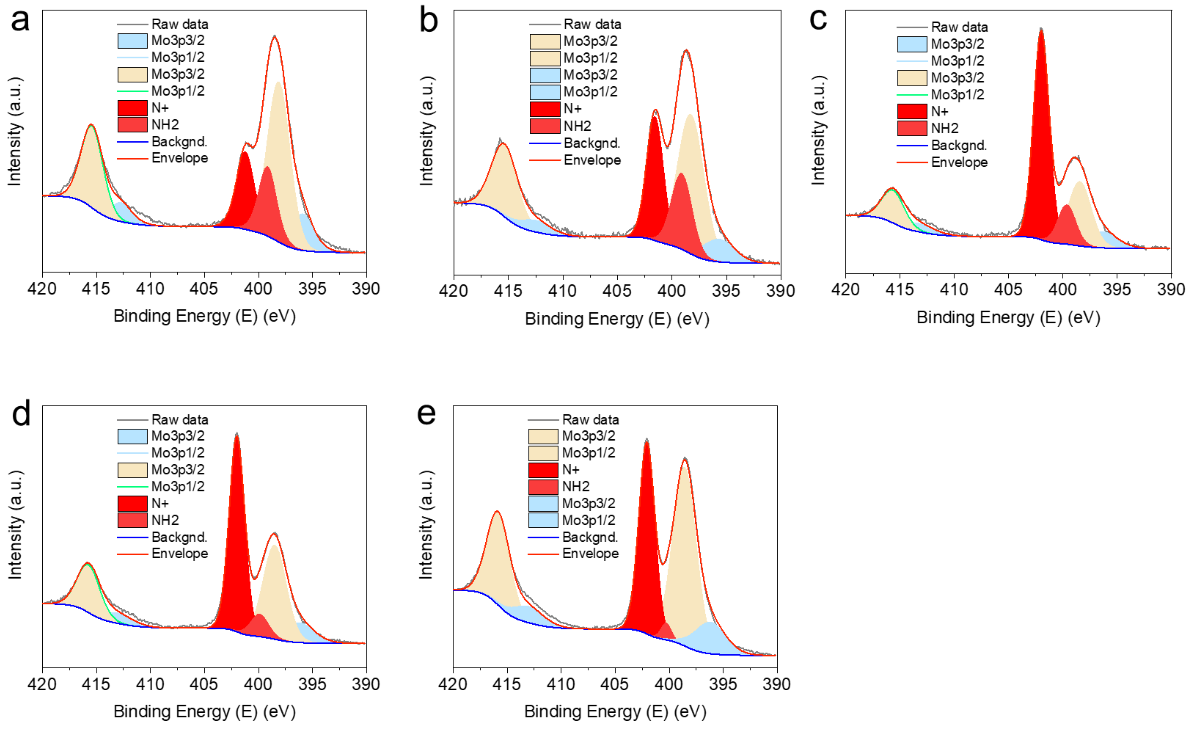

Figure S13. N1s and Mo3p peak fitting of pP-Mo precursors obtained from different acid ratios: a) pP-Mo-5/1, b) pP-Mo-1/1, c) pP-Mo-1/2, d) pP-Mo-1/3, and e) pP-Mo-1/4.

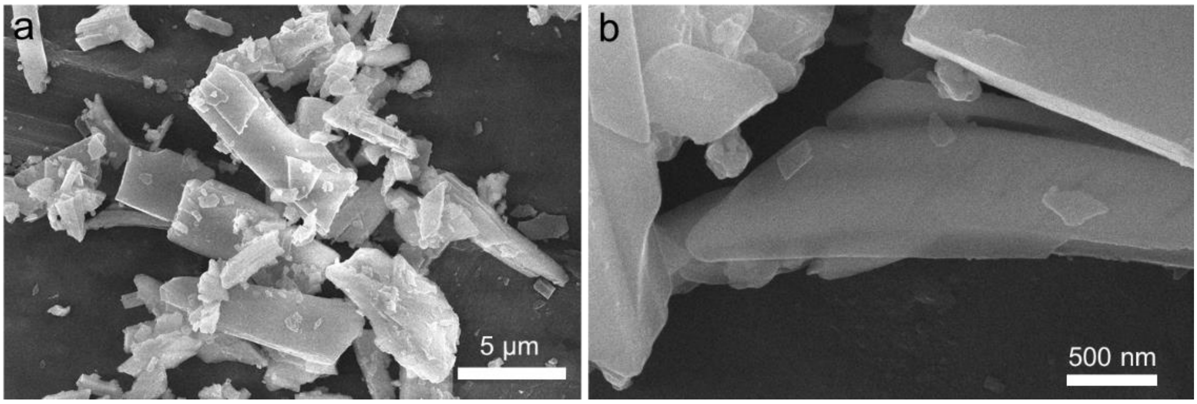

Figure S14. SEM images of a,b) pP-Mo-1/3 without silica nanoparticles.

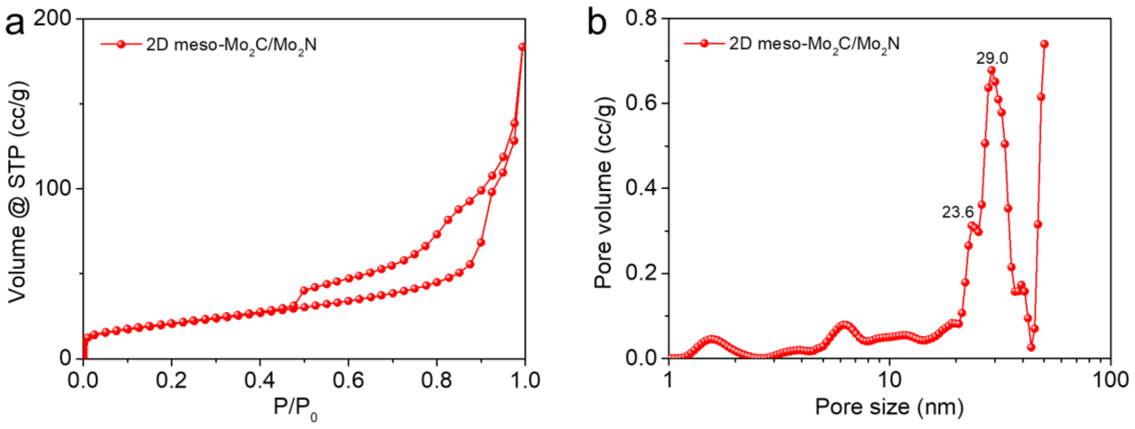

Figure S15. a) N_2 adsorption/desorption analysis of the 2D meso-Mo_2C/Mo_2N, and b) corresponding pore size distribution, which was calculated from the adsorption branch of the isotherms by the QSDFT model for cylinder/sphere pores.

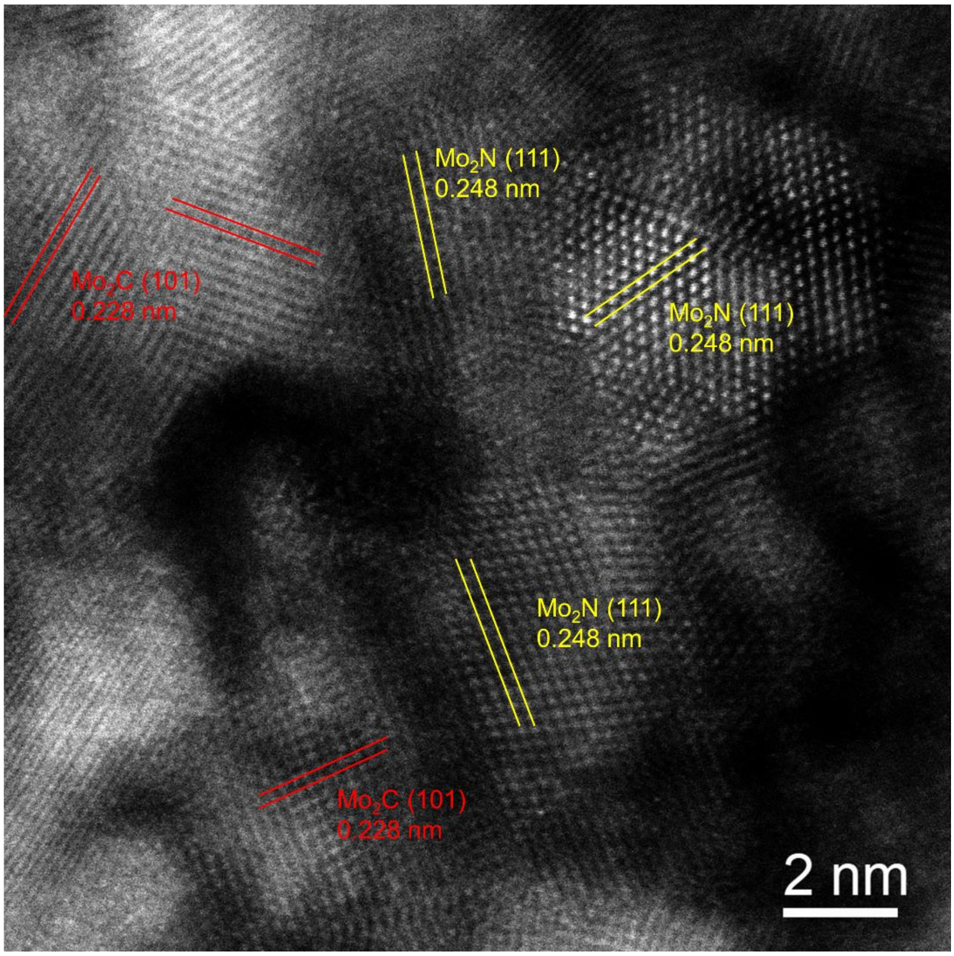

Figure S16. High-resolution HAADF-STEM image of the 2D meso-Mo_2C/Mo_2N.

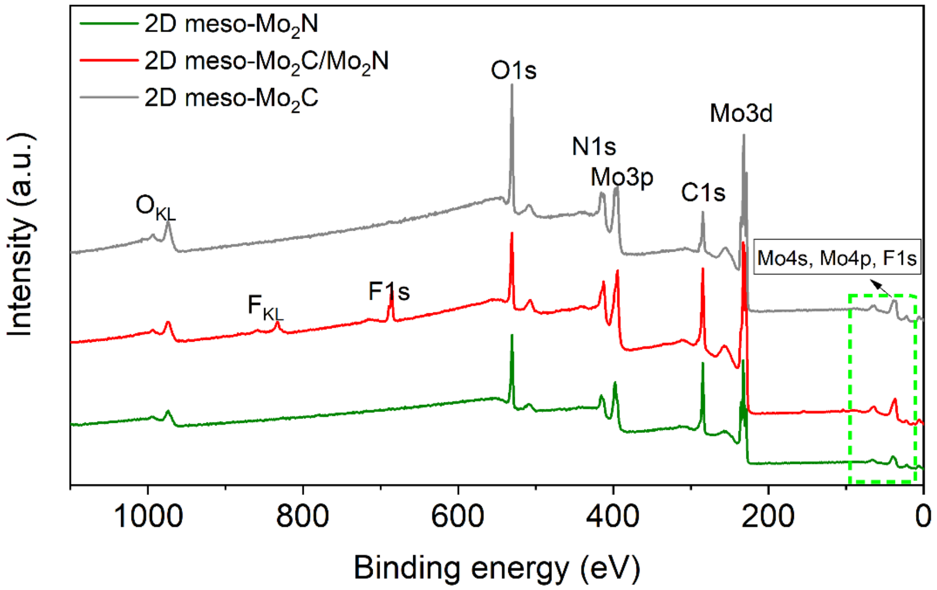

Figure S17. XPS survey spectra of the 2D meso-Mo_2N, 2D meso Mo_2C/Mo_2N, and 2D meso-Mo_2C. Note: the F1s peaks most probably stem from residues from the etching of silica with NH_4HF_2 solution.

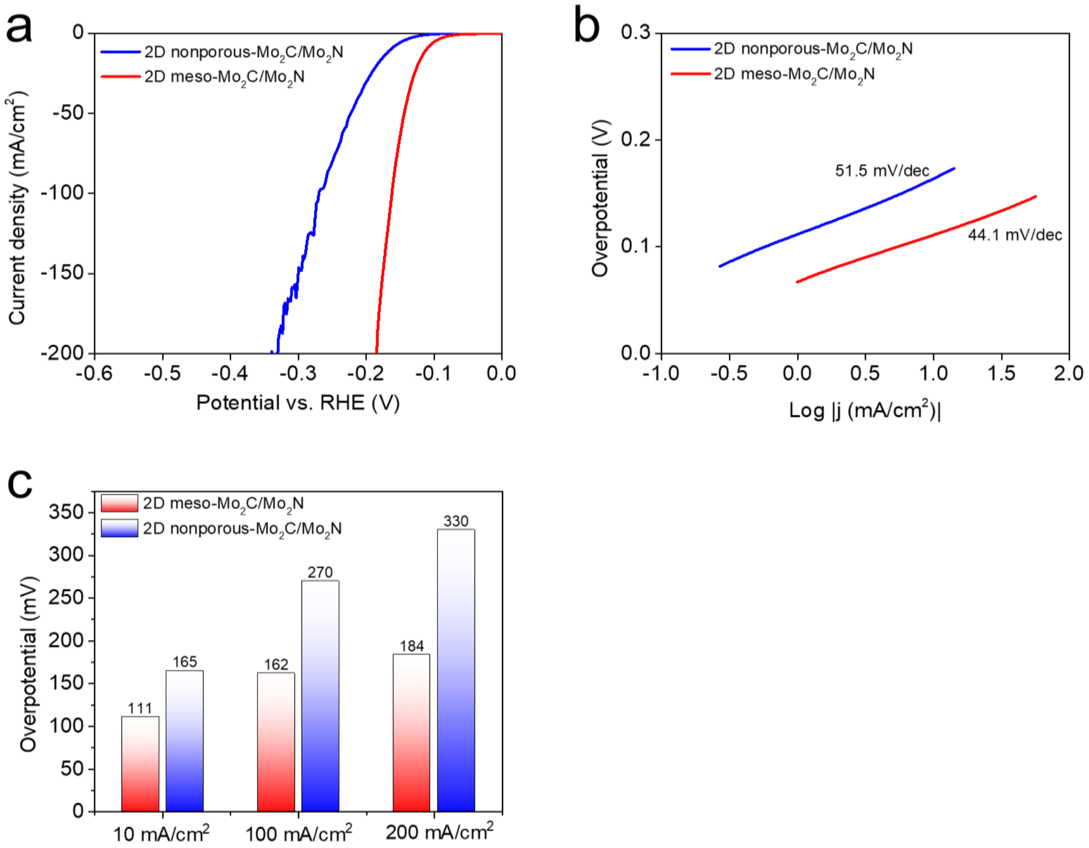

Figure S18. a) Polarization curves, b) Tafel plots, and c) overpotential at the different current densities of 2D meso-Mo_2C/Mo_2N and nonporous catalysts.

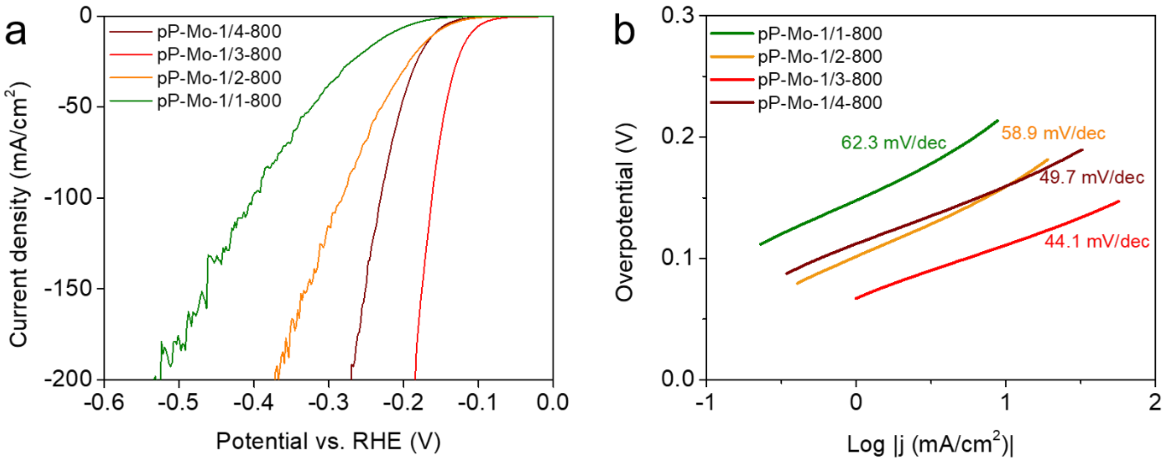

Figure S19. a) Polarization curves, b) Tafel plots of different catalysts synthesized from the pP-Mo precursors with different amounts of acid.

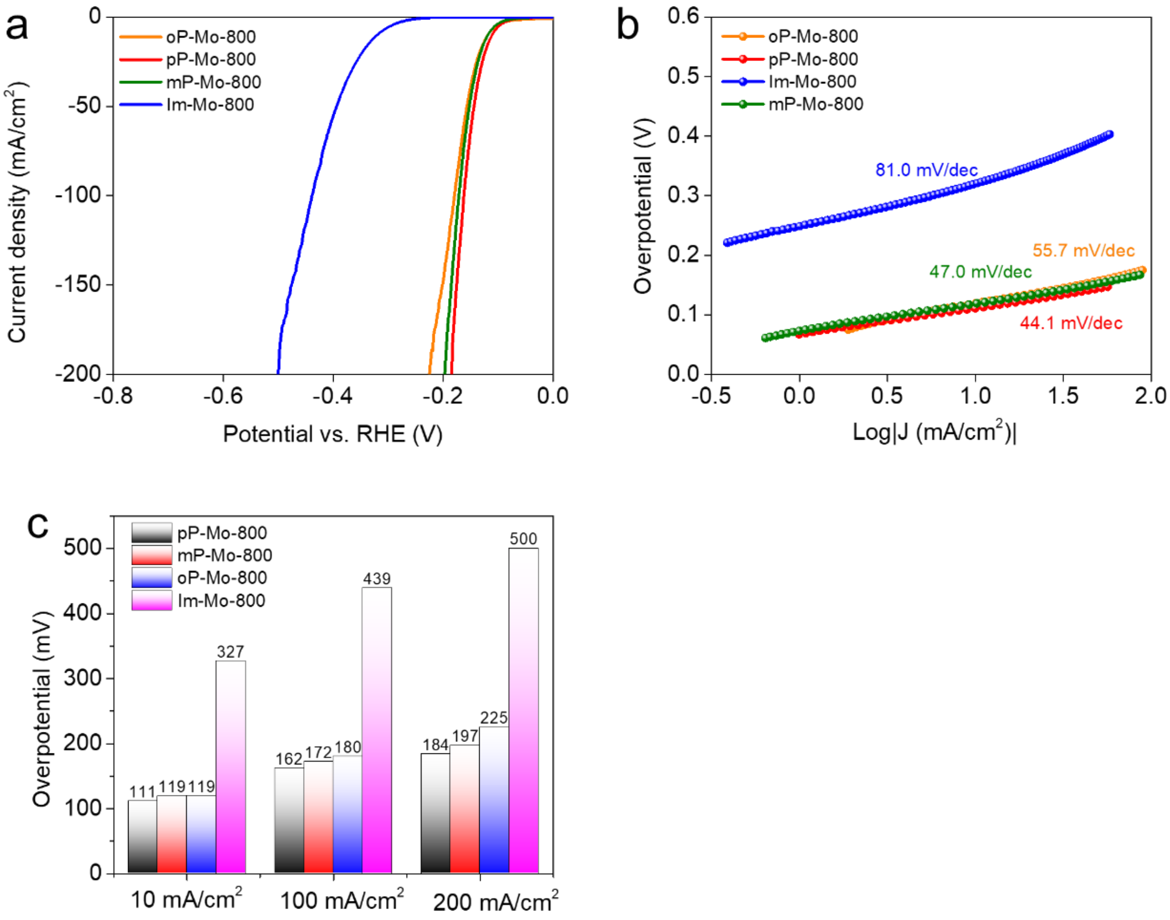

Figure S20. a) Polarization curves, b) Tafel plots, and c) overpotential at the different current densities of 2D meso-Mo_2C/Mo_2N synthesized from different precursors synthesized with different organic molecules (pP, mP, oP, and Im).

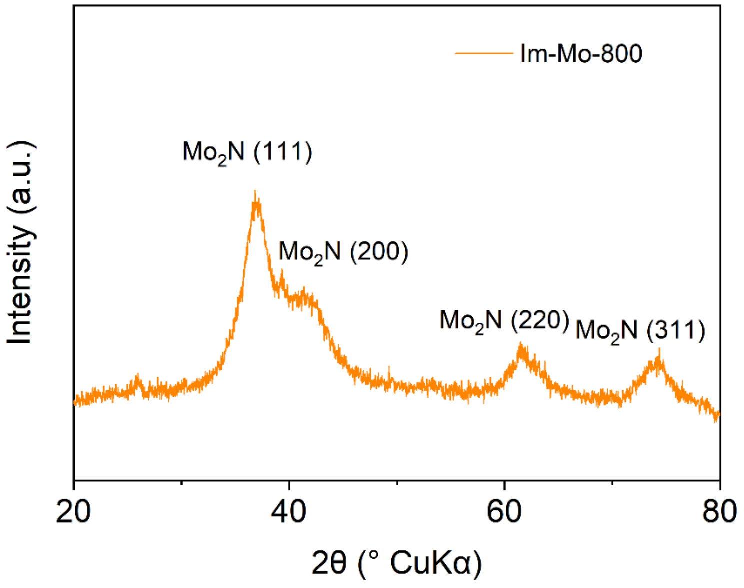

Figure S21. XRD pattern of Im-Mo-800

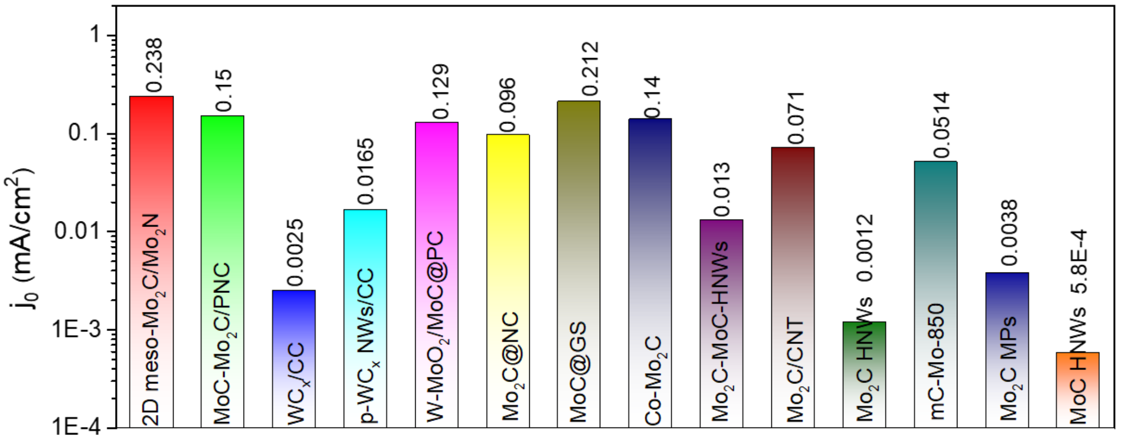

Figure S22. Exchange current density of the 2D meso-Mo_2C/Mo_2N and recently reported results.

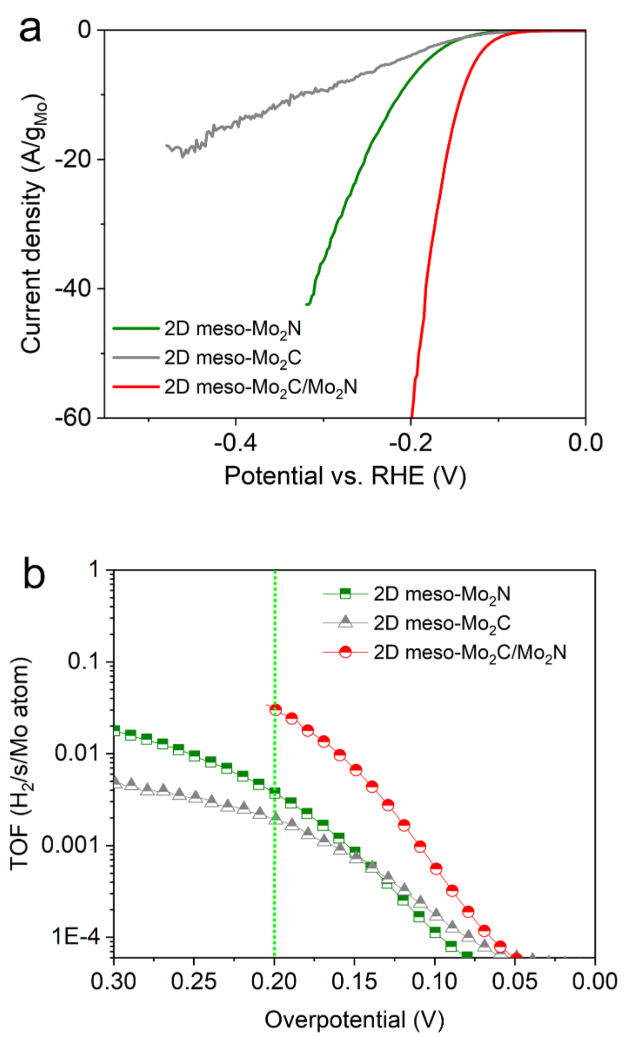

Figure S23. a) mass activity based on the weight content of Mo; b) TOF values of the catalysts based on Mo atoms.

Table S1. Elemental content of C, N, O, Mo obtained from XPS

	C (wt.%)	N(wt.%)	O(wt.%)	Mo(wt.%)
**2D meso-Mo_2N**	29.7	8.8	16.6	44.9
**2D meso Mo_2C/Mo_2N**	27.3	3.3	12.2	57.2
**2D meso-Mo_2C**	15.6	1.5	20.3	62.6

Table S2. Summary of recently reported representative HER electrocatalysts in seawater.

Catalyst	η10 (mV)	η50 (mV)	Tafel	Ref
**2D meso-Mo_2C/Mo_2N**	341	487	165	This work
**U-CNT-900**	~680			1
**CoSe**	330			2
**Mn-NiO-Ni/Ni-F**	~185			3
**CoMoP@C**	~450			4
**Mo_2C-MoP NPC/CFP-800**	346		173	5

References:

[1] Gao, S.; Li, G.–D.; Liu, Y.; Chen, H.; Feng, L.–L.; Wang, Y.; Yang, M.; Wang, D.; Wang, S.; Zou, X., Electrocatalytic H2 Production From Seawater Over Co, N–Codoped Nanocarbons. *Nanoscale* **2015**, 7, 2306–2316.

[2] Zhao, Y.; Jin, B.; Zheng, Y.; Jin, H.; Jiao, Y.; Qiao, S.-Z., Charge State Manipulation of Cobalt Selenide Catalyst for Overall Seawater Electrolysis. *Adv. Energy Mater.* **2018**, 1801926.

[3] Lu, X.; Pan, J.; Lovell, E.; Tan, T. H.; Ng, Y. H.; Amal, R., A Sea–Change: Manganese Doped Nickel/Nickel Oxide Electrocatalysts for Hydrogen Generation From Seawater. *Energy Environ. Sci.* **2018**, 11, 1898–1910.

[4] Ma, Y.–Y.; Wu, C.–X.; Feng, X.–J.; Tan, H.–Q.; Yan, L.–K.; Liu, Y.; Kang, Z.–H.; Wang, E.– B.; Li, Y.–G., Highly Efficient Hydrogen Evolution From Seawater by a Low–Cost and Stable CoMoP@C Electrocatalyst Superior to Pt/C. *Energy Environ. Sci.* **2017**, 10, 788–798.

[5] T. Liu, H. Liu, X. Wu, Y. Niu, B. Feng, W. Li, W. Hu and C. Li, *Electrochim. Acta*, **2018**, 280, 710-716.

Author Contributions

S. Li and A. Thomas conceived and designed the experiments. S. Li synthesized the materials and performed catalytic tests. Z. Zhao did the modulation of the Mo_2C/Mo_2N structure, construct and optimize the heterojunction modules. S. Li, P. Pachfule, and T. Ma discussed and solved the single crystal structure together. S. Li and A. Thomas provide the funding support, project administration, and wrote the whole paper together.
